# Comprehensive Analysis Based on the Cancer‐Immunity Cycle Identifies a Novel Immunosuppressive Subtype of Bladder Cancer

**DOI:** 10.1155/ijog/9720283

**Published:** 2026-04-06

**Authors:** Zexun Deng, Yifan Li, Yuyong Shen, Ming Zhou

**Affiliations:** ^1^ Department of Urology, The Affiliated Hospital of Yangzhou University, Yangzhou University, Yangzhou, Jiangsu, China, yzu.edu.cn

**Keywords:** BIRC5(), bladder cancer (BLCA), cancer-immunity cycle, exhausted immune class (EIC), immune checkpoint blockade (ICB)

## Abstract

**Background:**

Immune checkpoint blockade (ICB) has reshaped the approach to treating a broad spectrum of cancers, including bladder cancer (BLCA). However, a substantial fraction of BLCA patients still exhibit resistance despite high programmed death‐ligand 1(PD‐L1) expression in the tumor. Consequently, it is imperative to characterize the tumor immunosuppressive microenvironment (TME) and ascertain key biomarkers associated with immune evasion in order to stratify patients according to their propensity for immunotherapy resistance.

**Methods:**

A retrospective analysis of RNA sequencing data sourced from 403 BLCA samples was conducted during the course of our experimental research. Our research team employed weighted gene coexpression network profiling (WGCNA) and machine learning models to uncover tumor‐immune cycle‐associated genes that are tightly linked to BLCA. Unsupervised clustering was implemented to explore the tumor microenvironment‐associated gene expression profiles. Subsequently, in turn, we drew correlations between these expression signatures and a group of T cell exhaustion indicators, receptors, and immunotherapy responsiveness to further categorize them. Finally, a set of experiments were executed to verify the putative role of the core gene BIRC5 in BLCA at the experimental level.

**Results:**

Through comprehensive analysis, BIRC5, HSPA12A, and CXCL12 were identified as hub genes. Additionally, based on the molecular expression profiles of these three hub genes, BLCA patients were stratified into distinct subgroups. Simultaneously, this classification was correlated with the immune microenvironment landscape and established the exhausted immune class (EIC)—a novel subtype with high PD‐L1 expression, exhausted T cell markers, and immunosuppressive cytokines—in BLCA patients. Finally, we confirmed that BIRC5 exhibited high expression levels in BLCA and was involved in modulating the malignant proliferation, migratory activity, and invasive behavior of BLCA cells in vitro.

**Conclusion:**

The investigative group validated the oncogenic activity of BIRC5, a gene correlated with the tumor‐immune cycle. Additionally, our research team has identified a novel molecular subtype of BLCA that exhibits high expression of PD‐L1 but may potentially exhibit resistance to ICB therapy. These findings offer valuable insights into comprehensively characterizing the immunosuppressive tumor microenvironment in BLCA and present opportunities for deepened scrutiny of immunotherapy resistance mechanisms and optimization of targeted immunotherapeutic regimens.

## 1. Introduction

Bladder cancer (BLCA) is categorized among the most prevalent malignant neoplasms of the urinary system, particularly within middle‐aged and senior populations [1]. Although a range of treatment modalities have been established in recent years, including surgical resection and systemic chemotherapy, the majority of patients develop treatment‐related recurrence, leading to a poor health status and life functionality [2]. As the discipline of immune checkpoint blockade (ICB) immunotherapy has advanced, regulatory bodies have approved anti‐PD‐1/PD‐L1 inhibitors as an important treatment modality for BLCA patients presenting with tumor PD‐L1 expression levels of ≥ 50% [3, 4]. ICB has played a revolutionary role in the treatment of BLCA. However, there is still a significant proportion of BLCA patients who are not sensitive to this regimen, despite high PD‐L1 expression in the tumor [5]. Therefore, it becomes essential to define the tumor immunosuppressive microenvironment (TME) and screen for biomarkers linked to immune escape, thereby predicting immunotherapy resistance in affected patients.

Dubbed the cancer‐immunity cycle (CIC), it constitutes a step‐by‐step physiological process wherein the human immune system initiates a defensive reaction against tumor‐specific antigens and eradicates neoplastic lesions; Chen and Mellman initially proposed this conceptual model in 2013 to uncover how the immune system exerts antitumor cytotoxicity [6]. The CIC comprises seven core phases. Phase 1: Liberation of tumor‐associated antigens (neoplastic cell death); Phase 2: Tumor antigen presentation (dendritic cells/APCs); Phase 3: T cell priming and activation (APCs–T cell interactions); Phase 4: T cell trafficking to tumors (mainly CTLs); Phase 5: T cell infiltration into tumors (CTLs and endothelial cells); Phase 6: T cell recognition of malignant cells (CTL–tumor cell contact); Phase 7: Tumoricidal elimination of cancer cells (immune–neoplastic cell interplay). Through this cycle, the host’s immune system is able to efficiently eliminate malignant tumor cells. However, neoplastic cells possess robust immune evasion capacity; they can circumvent immune surveillance through intrinsic cell alterations or TME remodeling, thus sustaining unregulated proliferations [7]. This phenomenon is called immune escape. Immune escape of tumor cells is essentially achieved by disrupting certain steps. Conversely, scientists have also proposed tumor immunotherapy, which augments or activates the body’s CIC, enabling the host’s immune system to eradicate cancerous cells and reach core clinical treatment endpoints. Antibody therapy targeting PD‐1 is a kind of tumor immunotherapy, and the molecular mechanism behind it is closely related to CIC. Nonetheless, a major fraction of affected individuals demonstrate refractoriness to such immunotherapeutic strategies. Conversely, ICB can induce distinct toxicities and concomitant immune‐related adverse events via immune response potentiation. Accordingly, exploring the vital association between the CIC and immunotherapeutic response is essential, so as to provide more valuable prediction methods and therapeutic targets for immune response.

In our study, we evaluated the molecular characterization of CIC‐related genes (CICGs) for the first time in BLCA. CICGs closely related to BLCA were screened out by combining multiple machine learning methods, and a reliable prognostic model based on them was constructed. Moreover, the TCGA‐BLCA cohort was categorized according to these key CICGs; we thoroughly clarified the correlation between CICGs and BLCA’s TME as well as immune evasion and provided innovative therapeutic targets and strategies for immunotherapy‐resistant BLCA cases.

## 2. Materials and Methods

### 2.1. Data Collection

Altogether 178 CICGs were obtained from the Tracking Tumor Immunophenotype database (https://biocc.hrbmu.edu.cn/TIP/index.jsp) [8]. Clinical information and gene expression data pertaining to BLCA patients were obtained from the TCGA database (https://portal.gdc.cancer.gov/). The org.Hs.eg.db annotation platform (a Bioconductor package) was utilized to transform each sample’s Entrez gene ID into the matching gene symbol, with the average value adopted when a single Entrez gene ID was covered by multiple probes.

### 2.2. Identification of Differentially Expressed CICGs

The Wilcoxon rank sum nonparametric assay was implemented to pinpoint differentially expressed CICGs (DECICGs) between normal tissues and BLCA in both the TCGA‐BLCA dataset and GSE166716 dataset. Transcriptomic differential expression assays were carried out through the “limma” package in the R environment, and heatmaps were used to display the differential expression outcomes across the two datasets.

### 2.3. Weighted Gene Coexpression Network Analysis (WGCNA)

The “WGCNA” package [9] was deployed to construct a coexpression network for the identification of hub modules relevant to BLCA. First, a Pearson correlation matrix for all pairs of genes was constructed according to the formula a_mn_ = |c_mn_|^β^ (c_mn_ = Pearson’s correlation between gene m and gene *n*; a_mn_ = adjacency between gene m and gene *n*). Subsequently, the soft threshold exponent (parameter β) was computed to prioritize strong intergenic correlations and penalize weak gene correlations. A well‐matched β parameter was applied to construct the scale‐free coexpression network. In the final step, gene significance (GS) and module membership (MM) were determined; clinical feature‐correlated modules exhibiting the highest *R* ^2^ and meeting the threshold of *p* < 0.05 were selected, and hub DECICGs were acquired via cross‐matching DECICGs with genes from BLCA‐related modules.

### 2.4. Machine Learning

A trio of computational predictive learning frameworks was employed to filter hub DECICGs. Support vector machine recursive feature elimination is a reliable method for feature prioritization and discovery of key classification‐linked features, which is enacted with the “kernlab” and “e1071” toolkits. Random forest (RF) represents an ensemble‐based predictive learning approach implemented by the “randomForest” package to achieve classification via the establishment of a large set of decision trees. DECICGs that overlapped between WGCNA results and outputs from the three above machine learning assays were designated as hub genes. The “pROC” package was leveraged for establishing the receiver operating characteristic (ROC) curve and determining the area under the ROC curve (AUC), thereby gauging the discriminative capability of hub DECICGs to discriminate between BLCA and normal tissue. The clinical prediction accuracy of hub genes was evaluated by a nomogram and the calibration chart.

### 2.5. Cluster Analysis

Using the K‐means clustering algorithm, consensus clustering analysis was performed via the R package “ConsensusClusterPlus,” with 1000 replicate iterations conducted to obtain optimal clustering results. The “GSVA” R package was deployed to perform GSVA (gene set variation analysis) on gene profiles and compare variations in biological processes among separate clusters. GSVA is a nonparametric, unsupervised analytical approach for evaluating pathway perturbations or biological processes via stromal sample expression quantification. Gene collections retrieved from the molecular signature database were adopted as the standardized reference gene cohorts for subtype validation.

### 2.6. Identification of EIC

ESTIMATE was deployed to measure immune/stromal scores per BLCA case; CIBERSORT determined diverse immune cell subset abundances in all samples, and single‐sample gene set enrichment analysis (ssGSEA) analyzed immune cell functional properties linked to distinct BLCA molecular subtype. Eventually, the expression profiles of a panel of inhibitory receptors were analyzed, and the gene signature mimicking T cell exhaustion (TEX) was scored through the ssGSEA approach. Drawing on these results, the EIC was identified and selected.

Cluster 1, initially designated as the “immune–stromal cluster” based on high immune/stromal enrichment scores, was further defined as the EIC based on its distinct immunological features: high expression of exhausted T cell markers (PD‐1, CTLA‐4, LAG3, BTLA, TIGIT, TIM‐3, IDO1, SIGLEC7, VISTA), enrichment of immunosuppressive cytokines (IL‐10, TGF‐β, IL‐4), and elevated PD‐L1 expression.

### 2.7. Molecular Characterization of EIC

The Kaplan–Meier survival analysis approach and log‐rank assay were utilized to execute quantitative statistical contrasts across two distinct patient groups, with the core objective of gauging the prognostic implications of EIC. Jiang and colleagues elucidated the profiling and amelioration of T cell dysfunction [10]. Consequently, the research group of the present study evaluated and juxtaposed the predictive validity of the TIDE algorithm in terms of projecting the ICB immunotherapeutic response and clinical survival endpoints.

### 2.8. Clinical Specimens

BLCA tissues and matched peritumoral noncancerous tissues were obtained from surgically treated BLCA patients at the Affiliated Hospital of Yangzhou University (also known as Yangzhou No. 1 People’s Hospital) during the period of 2024–2025 (Table S1). The follow‐up cutoff was set as October 2025. All enrolled patients provided signed informed consent prior to clinical specimen use. The hospital’s Ethics Committee approved the application of biological specimens for this study.

### 2.9. Cell Culture and Transfection

Human T24 and UMUC3 cell lines were obtained from the Chinese Academy of Sciences Type Culture Collection (CAS‐TCCC, official abbreviation: CBTCCCA, Shanghai, China). T24 cells were cultured in the manufacturer‐recommended M5A culture medium, while UMUC3 BLCA cells were maintained in the experiment‐designated DMEM culture medium; both culture medium formulations were supplemented with 10% heat‐inactivated fetal bovine serum (FBS; BI).Cells were incubated in a humidified atmosphere containing 5% CO_2_ at 37°C.

For transfection of T24 and UMUC3 cells, BIRC5‐targeting siRNA sequences (chemically synthesized by Shanghai GenePharma, China) were used, with the company’s RNAi‐Mate reagent applied per the manufacturer’s protocols. The siRNA sequences targeting BIRC5 are provided in Table S2. Transfection was performed with 50 nmol of siRNA in six‐well plates using Lipofectamine 3000 when cells reached 50% confluence. BIRC5‐specific overexpression constructs and negative control plasmids were generated by GeneChem (Shanghai, China). A mammalian expression plasmid encoding flag‐tagged BIRC5 was constructed through molecular cloning, and its sequence was confirmed by DNA sequencing. Transfection with 1 μg of purified plasmid was performed when cells reached 80%–90% confluence. Subsequently, mRNA and protein levels in the cells were analyzed by real‐time quantitative PCR (qRT‐PCR) and Western blotting at 48 h and 72 h post‐transfection.

### 2.10. Cell Migration and Invasion Assays

Cell migration and invasion experiments were conducted using 24‐well culture plates fitted with transwell porous membrane modules (Corning, pore diameter: 8 μm). Approximately 1 × 10^4^ T24 urothelial carcinoma cells and 3 × 10^4^ UMUC3 urothelial carcinoma cells were resuspended in a 200‐μL serum‐starved culture medium and seeded into the upper compartment. The lower chamber was filled with 600 μL of medium supplemented with 10% FBS. T24 cells were incubated for a 24‐h period, whereas UMUC3 cells were maintained in incubation for 48 h. For migration assays, after the incubation period, the cells were subjected to fixation followed by staining with a crystal violet reagent (Beyobio Biotech). Transwell culture chambers for invasion assays were precovered with the Matrigel basement membrane matrix. Finally, microscopic images were obtained at magnification using an inverted light microscope (Nikon, Japan) for morphological evaluation.

### 2.11. Cell Viability Assay

Cell viability and proliferation capacity were evaluated using the Cell Counting Kit‐8 (CCK‐8)‐based method. Cells were initially resuspended, then diluted to a suitable concentration, and seeded into 96‐well culture plates at a seeding density of 1000 cells per well. These plates were subsequently placed in a humidified cell culture incubator maintained at 37°C with 5% CO_2_. For the proliferation assessment, 10 μL of CCK‐8 reagent was introduced into each well at 0, 24, 48, and 72 h, and the plates were incubated for an additional 2 h thereafter. The absorbance value at 450 nm was measured with a microplate reader (TECAN, Switzerland) to quantify cell viability and proliferation.

#### 2.11.1. Cell Colony Formation Assay

In vitro clonogenic survival assays were further implemented. A total of 1,000 T24 and UMUC3 cells were inoculated into individual wells of six‐well culture plates. After a 7‐day incubation period, culture supernatant was aspirated, and cellular monolayers were rinsed twice with sterile PBS. Cells were subsequently immobilized in 4% paraformaldehyde for 15 min, stained with crystal violet staining reagent, and subjected to microscopic imaging. Finally, observation and photography were conducted under an inverted optical microscope (Nikon, Japan).

### 2.12. Wound‐Healing Scratch Assay

The scratch wound‐healing experiment was adopted to gauge cell migratory competency. Cell suspensions were plated into six‐well cell culture plates 24 h ahead of the assay. The fully confluent cell layer was incised with a 200‐μL pipette tip, after which the cells were maintained in a serum‐starved culture medium for a 24‐h incubation period. Imaging of T24 cells was conducted at 0 h and 24 h, whereas UMUC3 cells underwent imaging at 0, 36, and 72 h. Microscopic visuals were obtained via an inverted biological microscope (Nikon, Japan).

### 2.13. qRT‐PCR and Western Blotting

Total cellular RNA derived from T24 and UMUC3 cell lines was extracted using TRIzol extraction reagent (Thermo Fisher Scientific, Waltham, USA). Target complementary DNA (cDNA) was synthesized by means of a commercial reverse transcription kit (Thermo Fisher Scientific, United States). qRT‐PCR assays were carried out using a LightCycler 480 II detection system (Roche Diagnostics, Basel, Switzerland), utilizing the SYBR‐Green Master Kit (Vazyme, Nanjing, China). Gene‐specific amplification primers were purchased from Qingke Biotech (Nanjing, China).

For total protein extraction, T24 and UMUC3 cells were subjected to lysis using the RIPA lysis buffer. Nuclear protein was purified through the nuclear protein extraction kit supplied by Biyuntian (Beijing, China). After blocking in the FastBlock blocking buffer (GBCBIO Technologies, Guangzhou, China) for 15 min, the nitrocellulose (NC) blotting membrane was subjected to overnight incubation at 4°C with primary antibodies that exhibited specific affinity for the proteins of interest. Subsequently, membranes were subjected to a 2‐h incubation with corresponding secondary antibodies.

### 2.14. Statistical Analysis

Pairwise comparisons were performed using the Wilcoxon rank sum test. Comparisons of distributions between three groups were made by the Kruskal–Wallis test (with the Dunn–Bonferroni post hoc correction method incorporated). The Kaplan–Meier survival estimator was employed to evaluate survival discrepancies between the low‐risk and high‐risk patient cohorts. Multivariate Cox proportional hazards regression analysis was carried out to screen out independent prognostic determinants for BLCA overall survival (BLCA‐OS). ROC curves were constructed to verify the predictive performance of the prognostic risk stratification model and clinical nomogram (statistical significance: *p* < 0.05). Primer sequences for BIRC5 were provided in Table S2.

## 3. Results

### 3.1. Identification of DECICGs

We first counted the coexisting CICGs in the TCGA‐BLCA cohort and the GSE166716 cohort, and finally, 166 CICGs were identified as widely expressed genes and applied to subsequent analysis (Figure 1(a)). We computed the set of DECICGs across tumor specimens and adjacent nontumor tissues within the TCGA‐BLCA dataset and GSE166716 gene expression cohort and visualized the expression patterns of these genes via hierarchical clustering heatmaps (Figures 1(b) and 1(c)). Ultimately, 11 CICGs were identified as DECICGs in both cohorts. Among them, ARG2, BIRC5, EZH2, and HSPA14 were upregulated in tumors, while CCL19, KLF2, HSPA12A, EDNRB, HSPA12B, HSPA2, and CXCL12 were downregulated in normal tissues (Figure 1(d)). To further demonstrate the prognostic relevance of these 11 DECICGs, a prognostic network map was constructed in TCGA‐BLCA cohort (Figure S1(a)). We found that the majority of DECICGs were risk factors for BLCA patients, including CXCL12, EZH2, EDNRB, HSPA2, CCL19, HSPA14, BIRC5, HSPA12B, HSPA12A, and KLF2. However, ARG2 and CCL19 were associated with favorable prognosis in BLCA patients. Survival analysis showed that CXCL12, EDNRB, KLF2, HSPA12B, and HSPA12A herald poorer OS of BLCA patients, with statistical significance (Figure S1(b)).

FIGURE 1Identification of DECICGs. (a) 166 CICGs were identified as coexisting CICGs in the TCGA‐BLCA cohort and the GSE166716 cohort. (b–c) Differentially expressed CICGs (DECICGs) between tumor and normal tissues in the TCGA‐BLCA cohort and GSE166716 cohort. (d) Eleven CICGs were identified as DECICGs in both cohorts. Red: upregulated genes; blue: downregulated genes.

**Figure:**
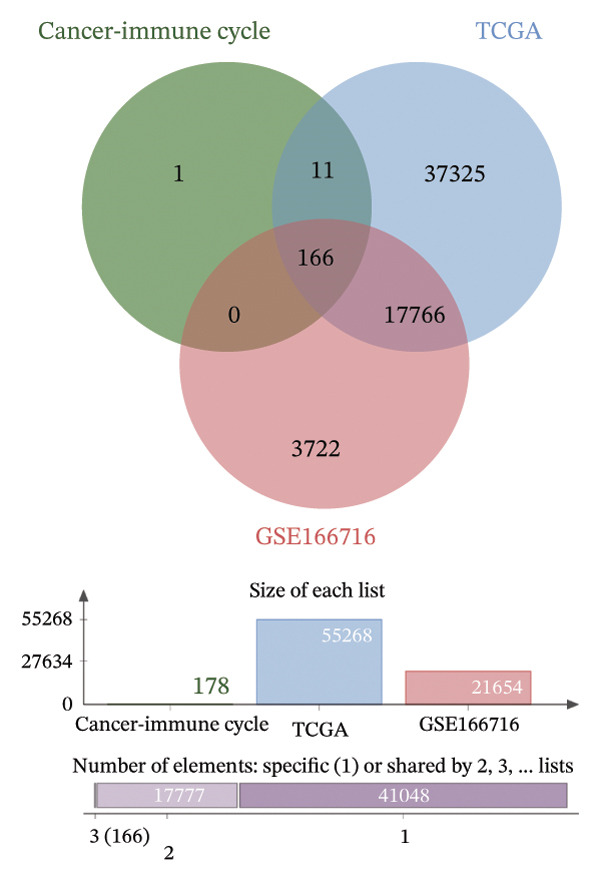
(a)

**Figure:**
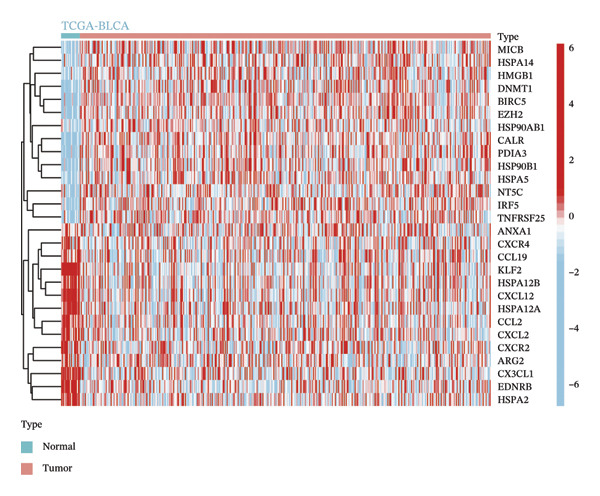
(b)

**Figure:**
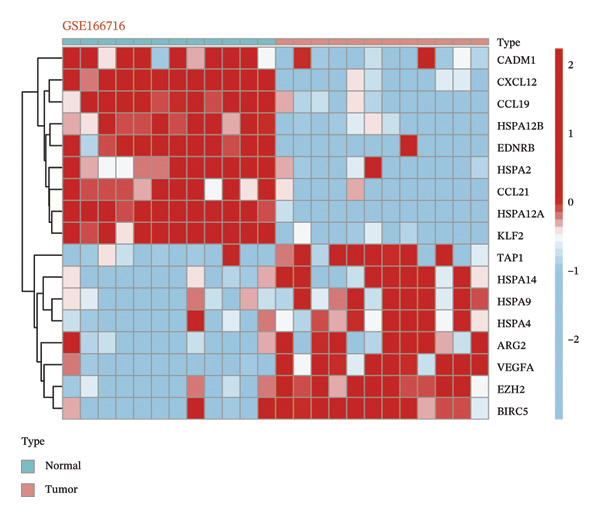
(c)

**Figure:**
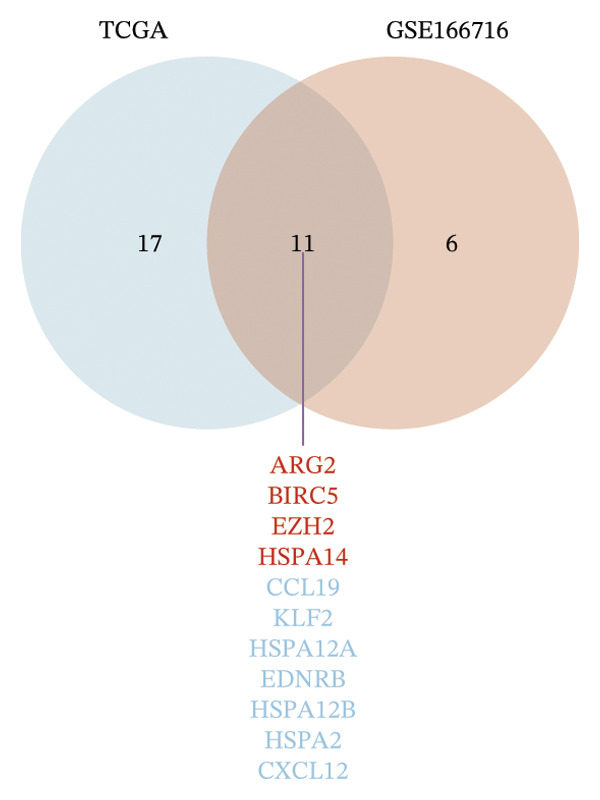
(d)

### 3.2. Screening of Hub DECICGs via WGCNA

The WGCNA algorithm was deployed to screen for gene modules tightly correlated with BLCA. Hierarchical clustering was performed on all specimens to eliminate outlier samples (Figure 2(a)). Subsequently, *β* = 6 was designated as the soft‐thresholding power value to ensure that the scale‐free *R* ^2^ attained a threshold of 0.9, with a scale‐free coexpression network subsequently established on this basis (Figure 2(b)). A hierarchical clustering dendrogram was generated, and gene modules were identified via the dynamic tree‐cutting algorithm (Figure 2(c)). Finally, a cumulative total of 20 distinct color‐labeled gene coexpression modules were delineated via WGCNA (Figure 2(d)). Of these identified gene modules, the blue module (*r* = 0.4, *p* < 0.001) and red module (*r* = 0.36, *p* < 0.001) exhibited significant positive correlations with BLCA. In contrast, the yellow module (*r* = −0.56, *p* < 0.001) and grey60 module (*r* = −0.31, *p* < 0.001) demonstrated notable negative correlations with BLCA (Figure 2(e)). Striking statistical associations were detected between module affiliation (MM) quantification values and gene relevance (GS) for genes integrated into the grey60 and yellow module clusters among nontumor tissue samples. Conversely, within BLCA tumor tissue specimens, distinct statistical associations were verified between MM quantification indices and GS relevance scores of genes assigned to the blue and red gene coexpression module subsets (Figure 2(f)). Subsequently, we extracted the genes of DECICGs and modules in BLCA positively and negatively correlated modules for crossover and found that BIRC5, EZH2, and HSPA14 were in the blue module, HSPA12A, HSPA1, and CXCL12 were in the yellow module, and BIRC5, EZH2, and HSPA14 were in the blue module. KLF2, EDNRB, and CHSPA12B are in the grey60 module (Figure 2(g)).

FIGURE 2(a) Hierarchical clustering of all samples excluding outliers. (b) *β* = 6 was set as soft‐thresholding power (scale‐free *R* ^2^ = 0.9) to construct a scale‐free coexpression network; left subpanel: scale‐free fit index vs. power (*x*‐axis, r‐axis corrected), right: mean connectivity vs. power. (c) Hierarchical clustering dendrogram was generated for module delineation. (d) Gene clustering dendrogram (dissimilarity from topological overlap) with module color labels; 20 color‐coded gene modules were identified. (e) Blue (*r* = 0.4, *p* < 0.001) and red (*r* = 0.36, *p* < 0.001) modules had positive correlations with BLCA; yellow (*r* = −0.56, *p* < 0.001) and grey60 (*r* = −0.31, *p* < 0.001) modules had negative correlations. (f) Scatter plots of GS (weight) vs. MM across modules; significant linkages between MM and GS were confirmed in blue/red modules. (g) Venn diagram showing intersection of DECICGs and WGCNA‐derived candidate genes.

**Figure:**
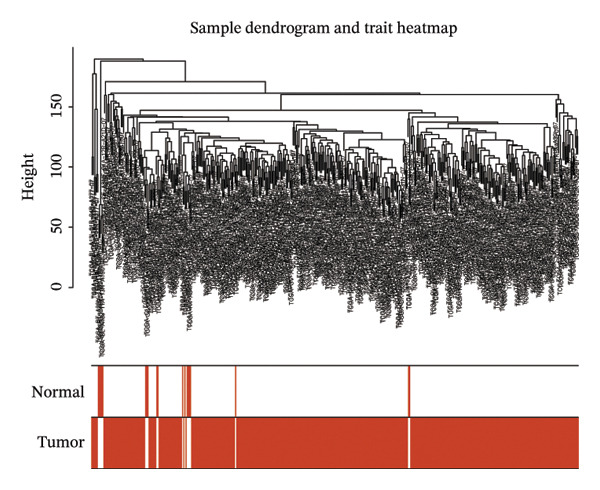
(a)

**Figure:**
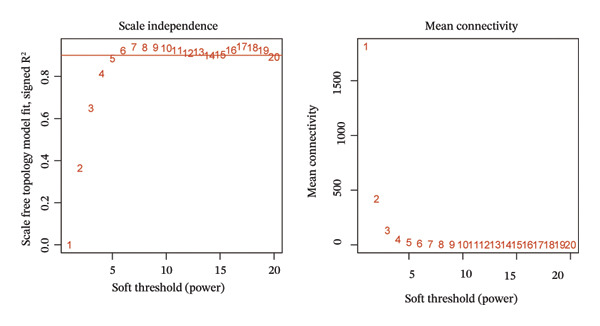
(b)

**Figure:**
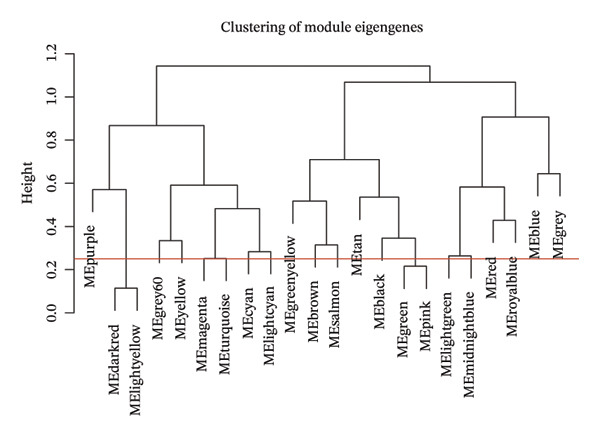
(c)

**Figure:**
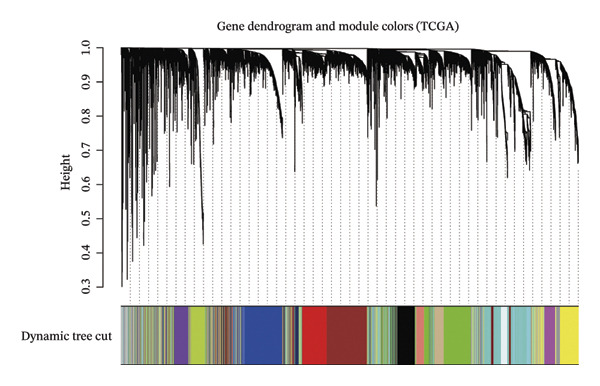
(d)

**Figure:**
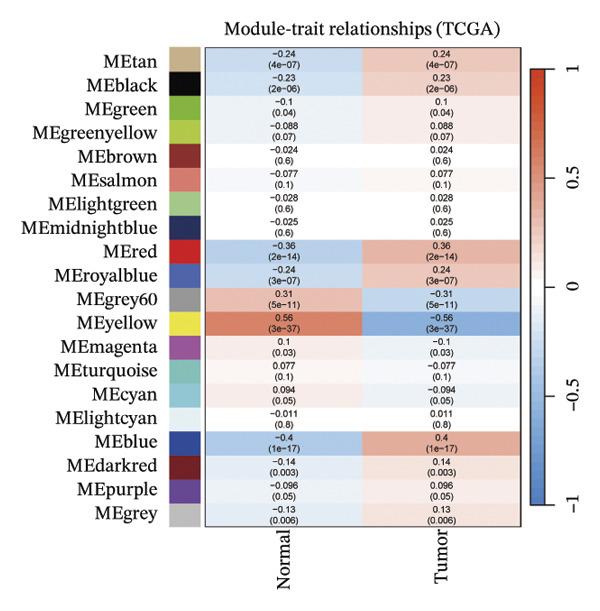
(e)

**Figure:**
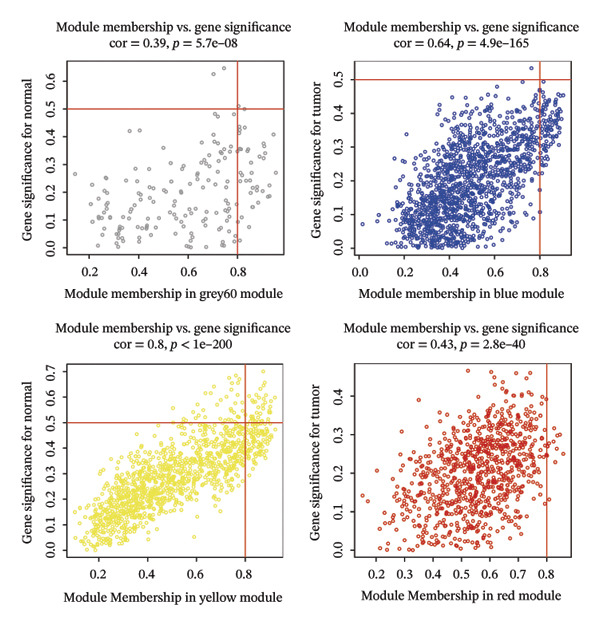
(f)

**Figure:**
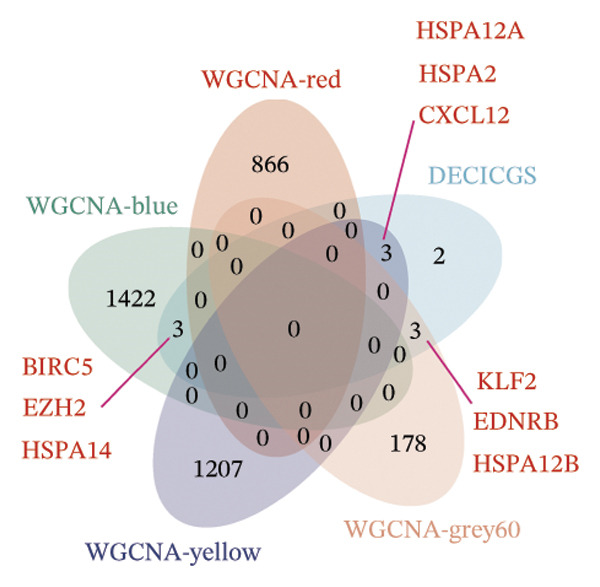
(g)

Validation was additionally performed in the GSE166716 cohort. The same analysis method as TCGA‐BLCA was applied again (Figures S2(a), S2(b), and S2(c). Finally, 12 color modules were identified in the GSE166716 cohort (Figure S2(d)). Among them, the green module (*r* = 0.79, *p* < 0.001) was positively correlated with BLCA, whereas the turquoise module (*r* = −0.95, *p* < 0.001) was negatively correlated with BLCA (Figure S2(e)). There was a significant correlation between the MM and GS of the turquoise module in normal tissues. In BLCA, MM was significantly correlated with the GS of the green module (Figure S2(f)). Similarly, intersection analysis showed that BIRC5 and EZH2 were present in the green module, while KLF2, HSPA12A, ARG2, EDNRB, HSPA2, CXCL12, and CCL19 were present in the turquoise module (Figure S2(g)).

### 3.3. Screening of Hub DECICGs via Machine Learning

Thereafter, potential BLCA‐associated DECICGs were screened for using three conventional machine learning approaches. First, the LASSO regression algorithm was employed to filter out overfitted genes among modular genes, seven of which were potential diagnostic biomarkers (HSPA12A, ARG2, BIRC5, HSPA2, CXCL12, HSPA14, and CCL19) (Figure 3(a)). Subsequently, eight genes (BIRC5, EZH2, KLF2, ARG2, EDNRB, HSPA12A, CXCL12, and HSPA2) from DEGs were obtained as potential diagnostic biomarkers by SVM‐RFE algorithm (Figure 3(b). In the RF algorithm, a total of 24 candidate genes with relative importance scores greater than 0.5 were identified, including EZH2, KLF2, CCL2, ARG2, CALR, BIRC5, SLAMF7, IL2, RAET1E, DNMT1, HSPA6, PDIA3, HSPA12A, ANXA1, CX3CL1, ITGB2, SLAMF1, EDNRB, CXCL12, HAVCR1, CD160, CD58, LAIR1, and CCR6 (Figure 3(c)). Combined with the results of WGCNA, we found that only three DECICGs (BIRC5, CXCL12, and HSPA12A) could be identified as hub genes of BLCA (Figure 3(d)). To evaluate the predictive performance of diagnostic biomarkers, ROC curve‐based diagnostic validation was implemented. Within the TCGA‐BLCA dataset, the AUC values for BIRC5, CXCL12, and HSPA12A attained 0.890, 0.906, and 0.867, respectively, indicating robust diagnostic predictive potential (Figure 3(e)). We also calculated the differential expression of these three hub genes in different BLCA tumor statuses, stages, and grades. It was found that high BIRC5 expression predicted tumor‐bearing status, higher stage, and higher grade. Conversely, CXCL12 and HSPA12A exhibited no statistically significant expression disparities across the distinct experimental subgroupings (Figures 3(f), 3(g), and 3(h)). The “timeROC” package was utilized to calculate time‐point‐specific AUC and cumulative survival, with subsequent plotting of time‐dependent AUC curves. The results showed that the AUC values for predicting OS (Figure 3(i)), DSS (disease‐specific survival) (Figure 3(j)), and progression‐free interval (PFI) metrics for the prognostic model (Figure 3(k)) of BIRC5 and HSPA12A were all higher than 0.5 at different time points, while CXCL12 had lower AUC values in all aspects. Finally, the nomogram and calibration curve were built via three hub genes; their BLCA survival predictions matched the actual observations with high concordance (Figures 3(l), 3(m)).

FIGURE 3Screening of hub DECICGs via machine learning. (a) LASSO regression algorithm to screen the overfitted genes in the modular genes. (b) SVM‐RFE algorithm. (c) RF algorithm. (d) Three DECICGs(BIRC5, CXCL12, and HSPA12A) could be identified as hub genes of BLCA. (e) The AUC values of BIRC5, CXCL12, and HSPA12A in the TCGA‐BLCA cohort. (f–h) The differential expression of these three hub genes in different BLCA tumor status, stage and grade. (i–k) The AUC values for predicting OS (overall survival), DSS (disease‐specific survival), and PFI (progression‐free interval). (l–m) A nomogram and its calibration curve based on the three hub genes (*p* < 0.05^∗^; *p* < 0.01^∗∗^; *p* < 0.001^∗∗∗^).

**Figure:**
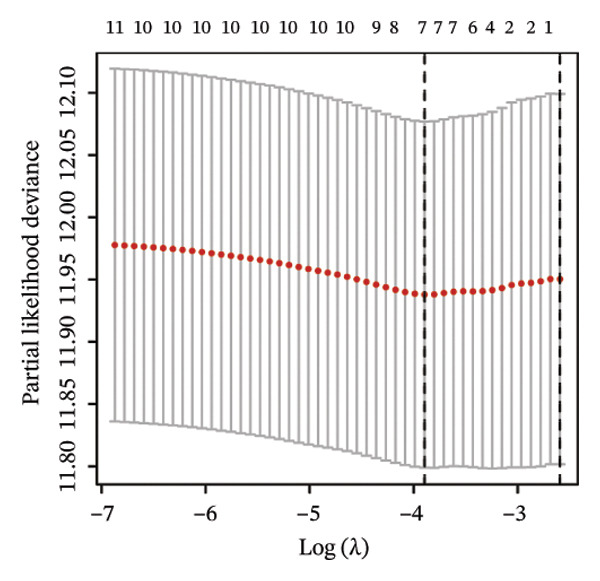
(a)

**Figure:**
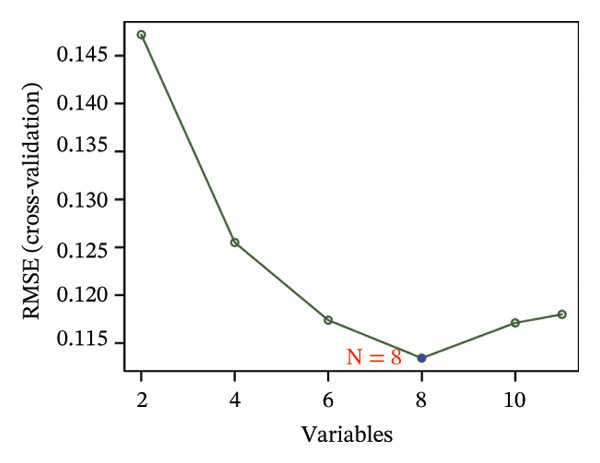
(b)

**Figure:**
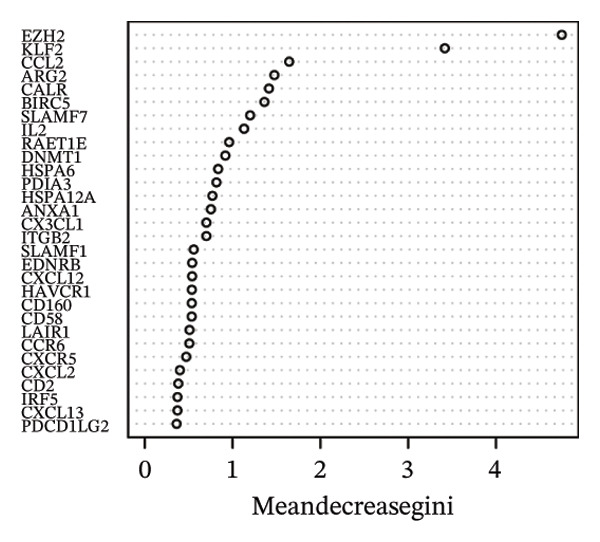
(c)

**Figure:**
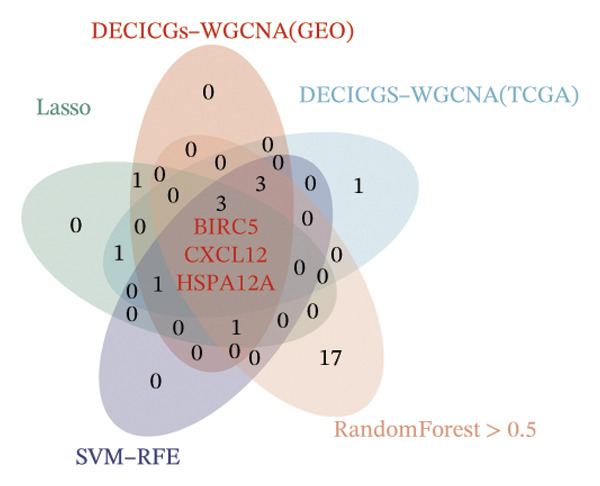
(d)

**Figure:**
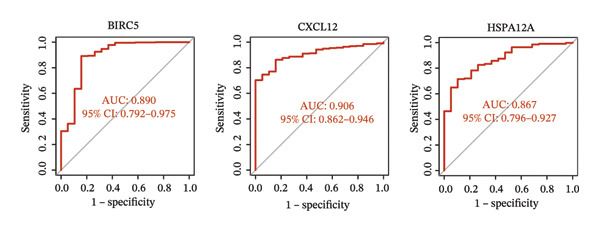
(e)

**Figure:**
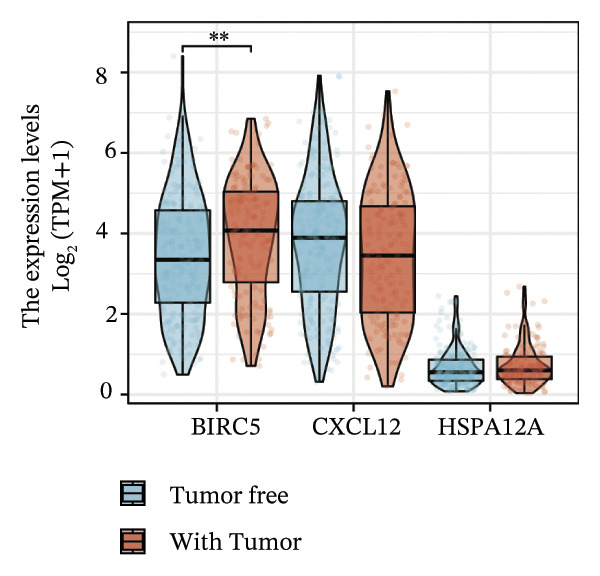
(f)

**Figure:**
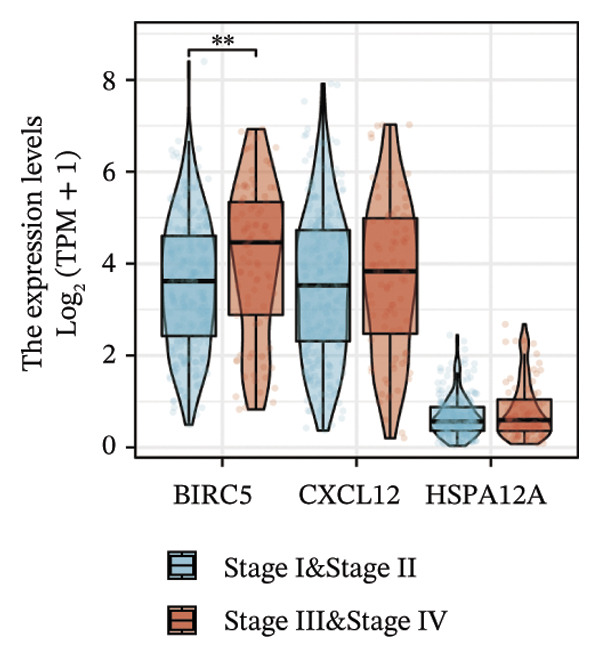
(g)

**Figure:**
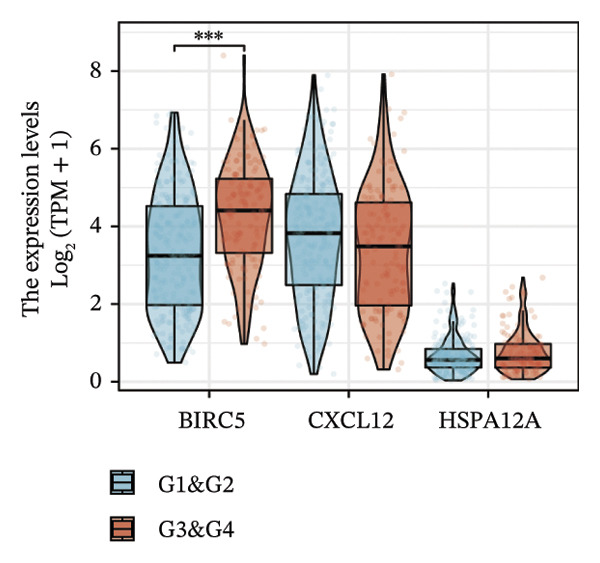
(h)

**Figure:**
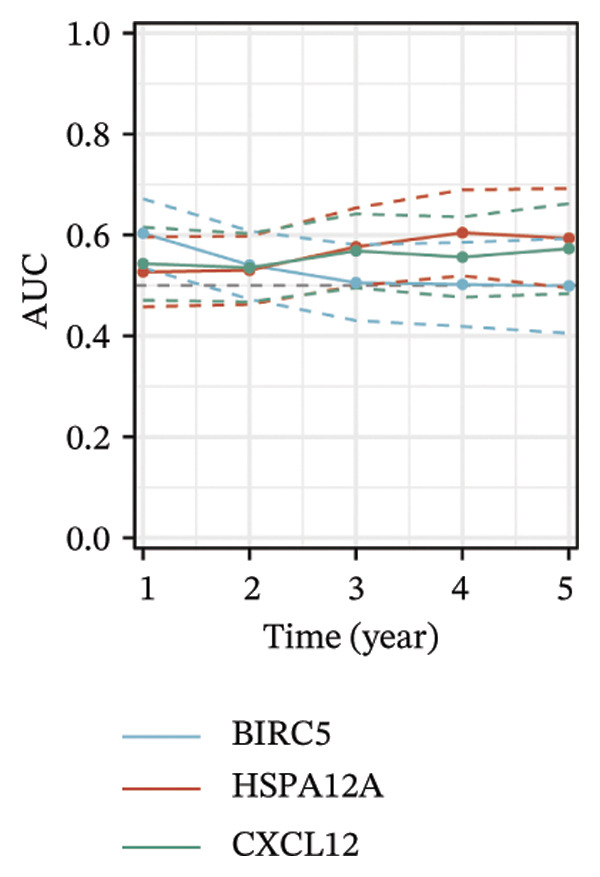
(i)

**Figure:**
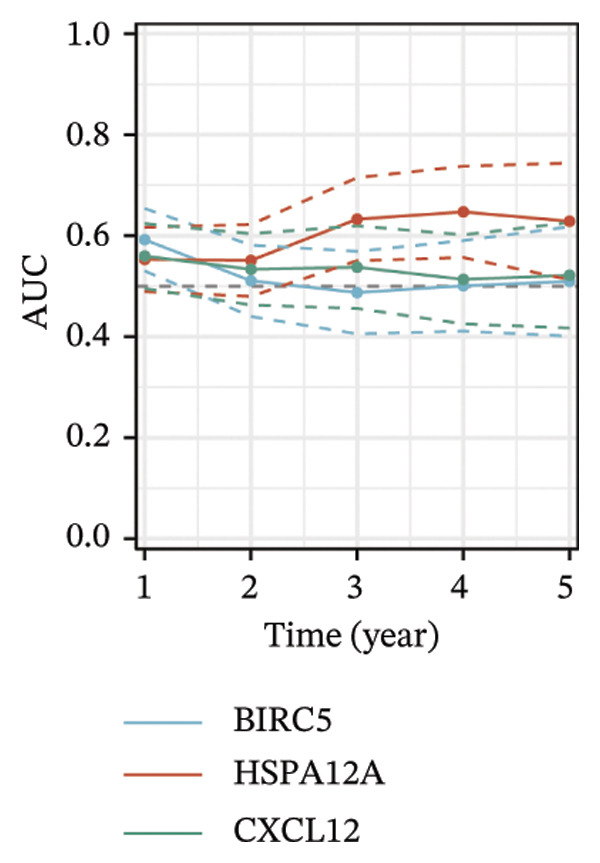
(j)

**Figure:**
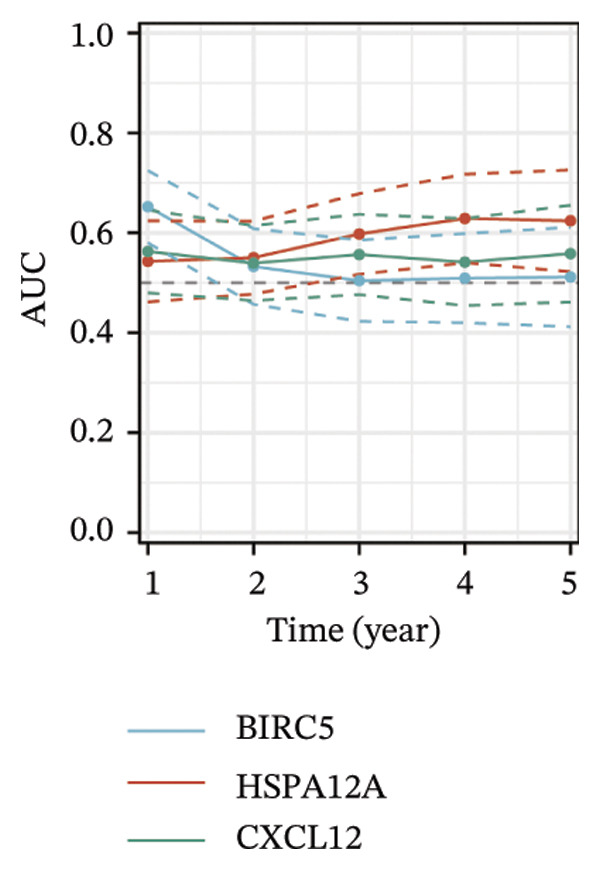
(k)

**Figure:**
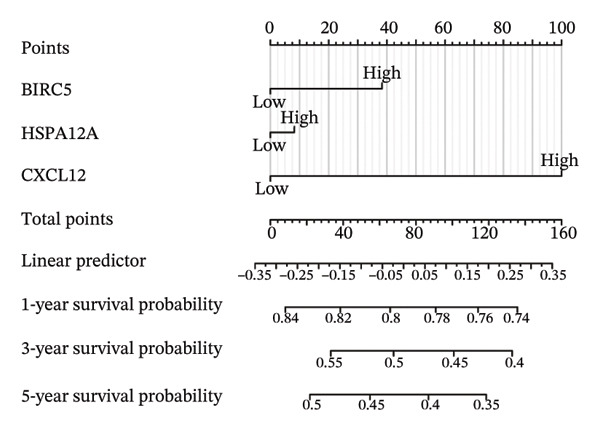
(l)

**Figure:**
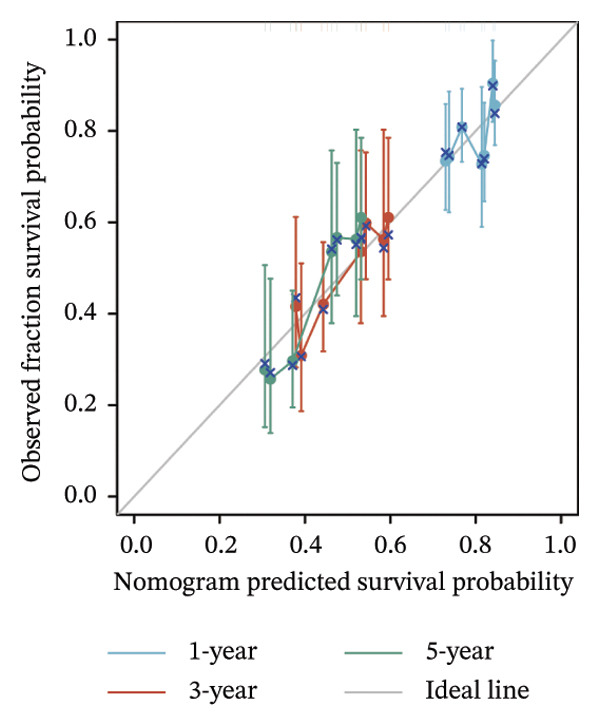
(m)

### 3.4. Identification of Three Clusters in TCGA‐BLCA Cohort Based on Three Hub Genes

Consensus hierarchical clustering assay was implemented for TCGA‐BLCA tumor tissue samples in accordance with genomewide gene expression profiles, and subtypes of BLCA were found through hub DECICGs analysis. When the value of cluster‐K is 3, the clustering result is relatively stable (Figure 4(a)). The consensus matrix and silhouette plots showed that the 403 TCGA‐BLCA samples could be divided into three different clusters, namely, Cluster 1 (*n* = 89), Cluster 2 (*n* = 261), and Cluster 3 (*n* = 53) (Figures 4(b) and 4(c)). The PCA clustering plot revealed distinct distribution patterns of the three clusters across the total sample, enabling clear discrimination (Figure 4(d)). Furthermore, survival analysis indicated that these three clusters corresponded to distinct survival outcomes in BLCA patients. Patients in Subtype 1 and Subtype 2 have poor survival expectations, whereas patients in Subtype 3 have better survival (Figure 4(e)). Furthermore, intercluster differential gene expression profiling analysis revealed that HSPA12A and CXCL12 exhibited markedly elevated expression levels within the Cluster 1 molecular subtype, whereas BIRC5 was highly expressed in Cluster 2. Subtype 3 has lower expression of all hub genes (Figure 4(f)). GSVA enrichment analysis demonstrated that distinct clusters were enriched in disparate biological pathways (Figures 4(g), 4(h), and 4(i)). Notably, Cluster 2 enriched cancer‐related pathways (cell cycle, DNA replication, and RNA polymerase).

FIGURE 4Identification of three clusters in the TCGA‐BLCA cohort based on three hub genes. (a) When the value of cluster‐K is 3, the clustering result is relatively stable. (b–c) Consensus clustering matrices and silhouette profile plots demonstrated that the 403 TCGA‐BLCA samples could be grouped into three distinct molecular subtype clusters. (d) The PCA clustering visualization portrayed the partitioning pattern of the three aforementioned clusters across the entire sample cohort. (e) Survival analysis of these three clusters. (f) Intercluster differential expression profiling revealed that HSPA12A and CXCL12 exhibited markedly elevated expression within Cluster 1, whereas BIRC5 was highly expressed in Cluster 2. (g–i) GSVA enrichment analysis (*p* < 0.05^∗^; *p* < 0.01^∗∗^; *p* < 0.001^∗∗∗^).

**Figure:**
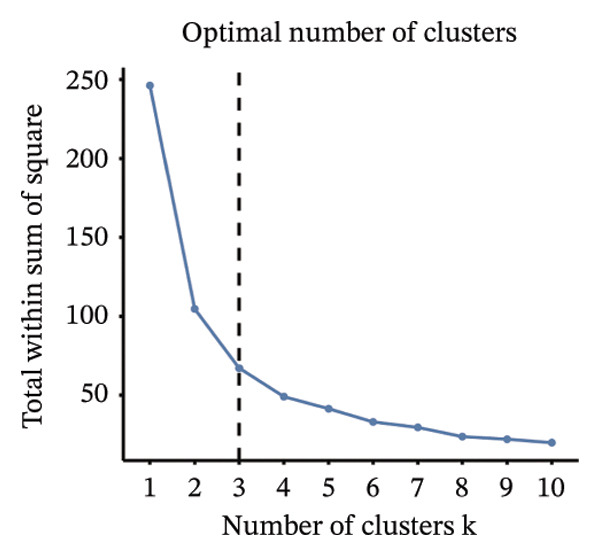
(a)

**Figure:**
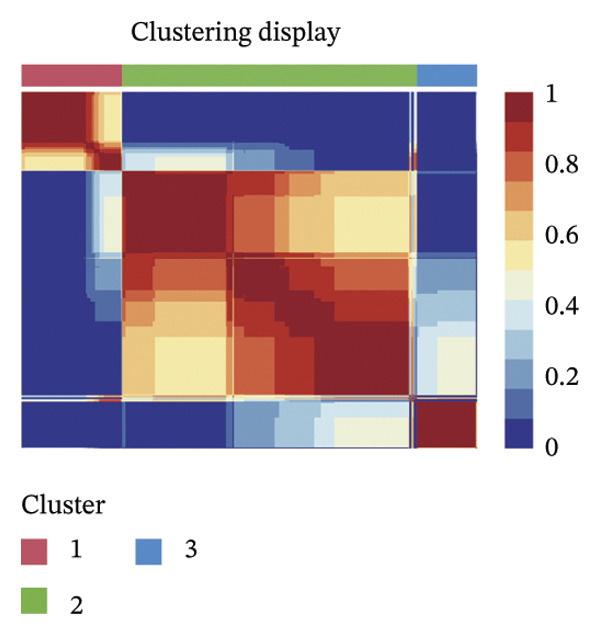
(b)

**Figure:**
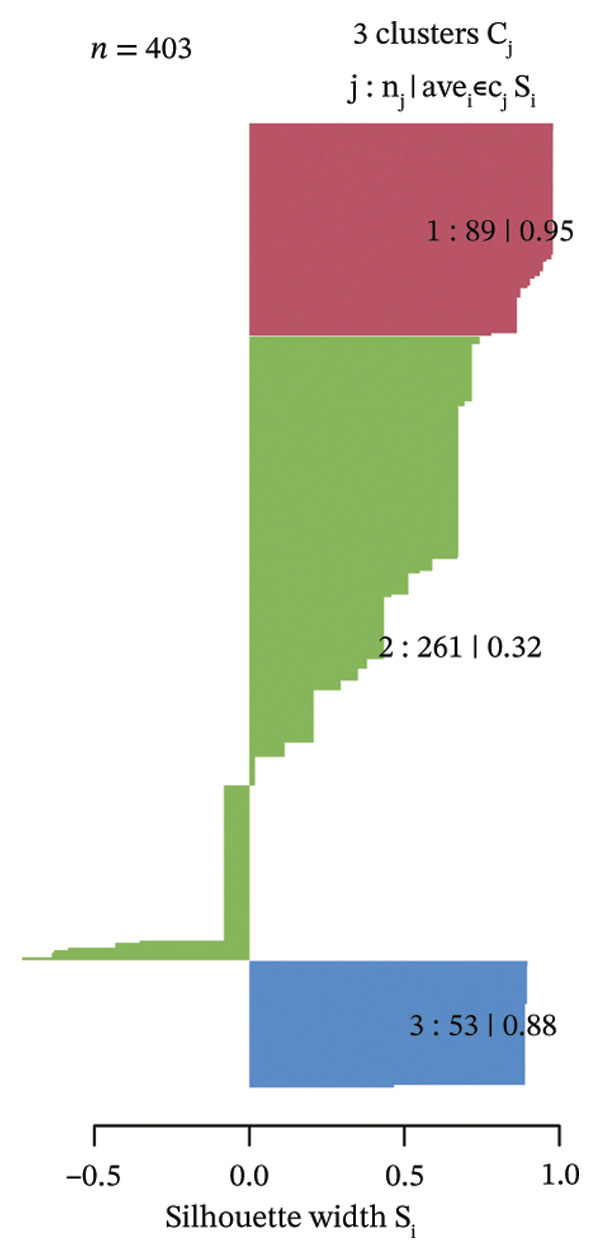
(c)

**Figure:**
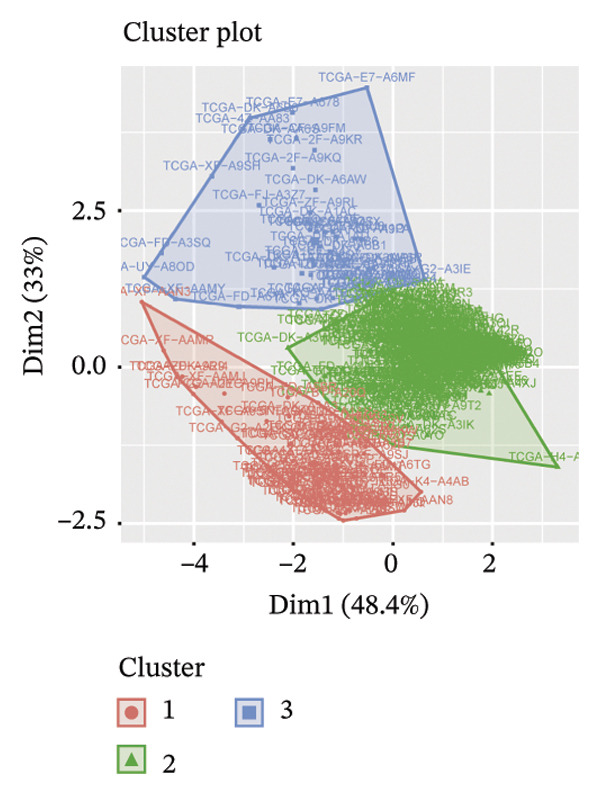
(d)

**Figure:**
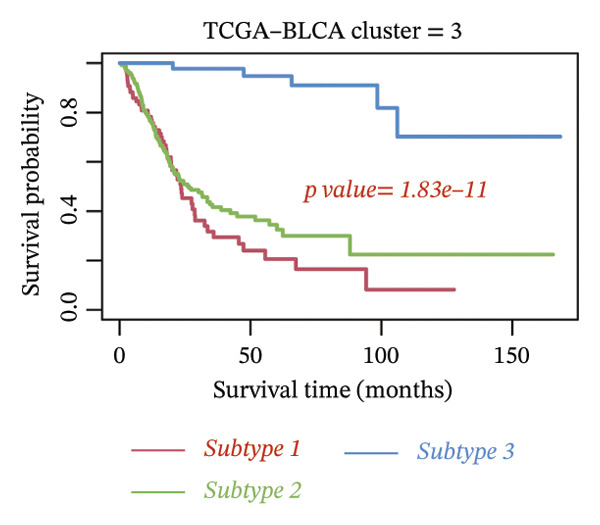
(e)

**Figure:**
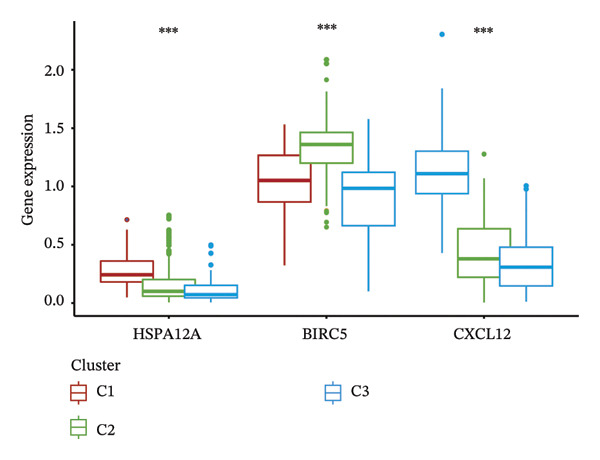
(f)

**Figure:**
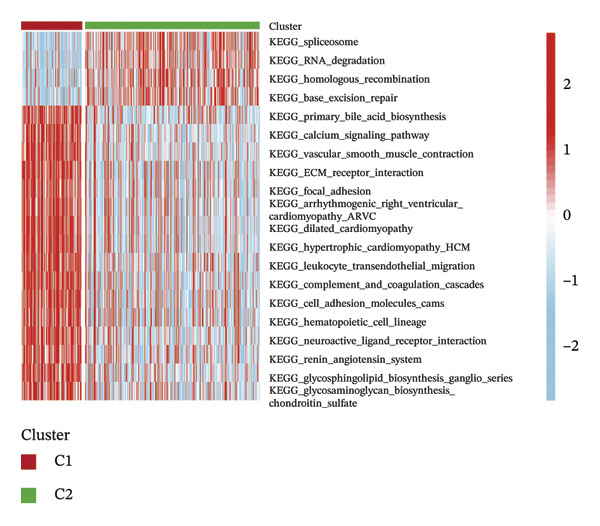
(g)

**Figure:**
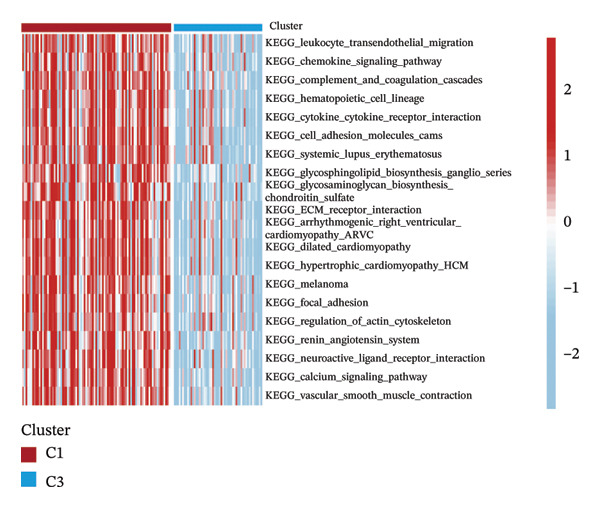
(h)

**Figure:**
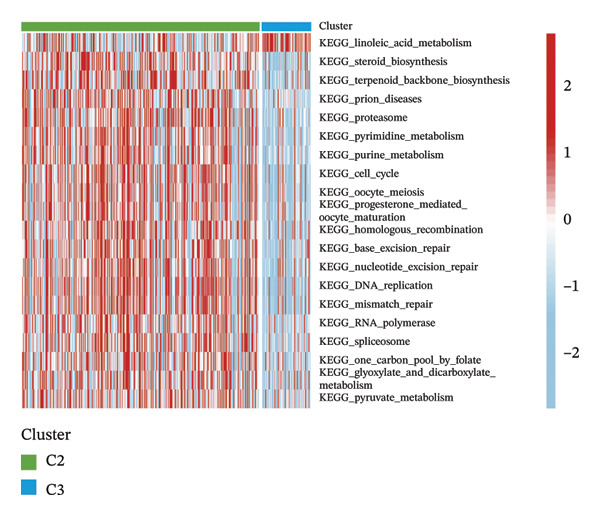
(i)

### 3.5. Identification of EIC

Immune and stromal infiltration enrichment indices were quantified via ssGSEA to delineate transcriptional expression profiles associated with immune and stromal components. Upon combining immune and stromal enrichment scores with the three subgroups derived from K‐means and consensus clustering, we found that cluster 1 exhibited higher enrichment scores relative to the remaining subgroups (Figure 5(a)). Therefore, this study identifies Cluster 1 as the “immune matrix cluster.” Then, we intended to look at the abundance of specific immune cells in tumors of the immune matrix cluster. We made use of signatures that represent different immune cells from published studies to calculate enrichment scores based on ssGSEA expression profiles. The immune–stromal cluster also exhibited distinct enrichment of immune cell‐associated signatures (Figure 5(b)). This cluster comprised key immune components (immune cell subgroups, immune signaling modulators, effector memory T cells (Tem), Th1/2 cell subsets, cytotoxic lymphocyte populations, T‐NK cell subsets, T/B cell lineages, macrophage subsets, and dendritic cell populations). Further confirmation of immune cell enrichment in the immune–stromal cluster involved contrasting RNA‐seq‐derived absolute proportions of immune cells across all clusters; consistent with ssGSEA findings, this cluster had greater levels of T cells, NK cells, M2 macrophages, and B cells than the remaining clusters (Figures 5(c), 5(d)). To unravel TEX functional phenotypical signatures in BLCA, global transcriptome characterization of key immune checkpoint repressive receptors (CTLA4, PDCD1/PD‐1, LAG3, BTLA, TIGIT, HAVCR2/TIM‐3, IDO1, SIGLEC7, and VISTA) was completed; the receptors displayed notable upregulation in immune–stromal cluster tumor isolates (fold change(FC) > 2, false discovery rate (FDR) < 0.05) (Figure 5(e)). Synchronously, clinical isolates assigned to the immune–stromal cluster displayed notable enrichment for a suite of TEX‐restricted gene sets (Figure 5(f)). Consequently, the immune–stroma‐linked cluster was additionally designated as the exhausted immune phenotype subtype (EIC).

FIGURE 5Identification of exhausted immune class. (a) ssGSEA to reveal immune and stromal‐related expression patterns. (b) Some signatures representing various immune cells from published studies were used to calculate enrichment scores based on ssGSEA expression profiles. (c–d) The relative absolute fractions of immune cell cohorts—ascertained via large‐scale RNA‐seq datasets—were contrasted between the immune–stromal cluster and other cluster cohorts. (e) Transcriptome profiling of a repertoire of immune checkpoint repressive mediators. (f) Patient‐derived clinical specimens stratified into the immune–stromal cluster unveiled pronounced enrichment for a repertoire of TEX‐defining gene signatures (*p* < 0.05^∗^; *p* < 0.01^∗∗^; *p* < 0.001^∗∗∗^).

**Figure:**
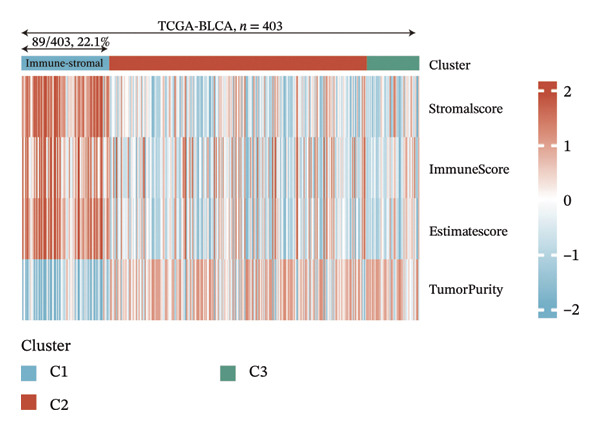
(a)

**Figure:**
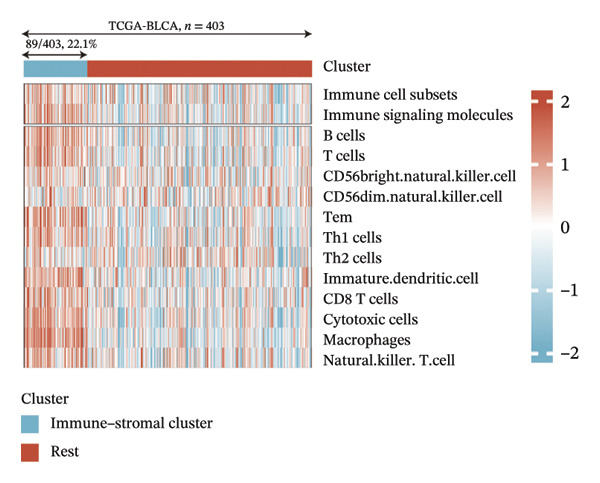
(b)

**Figure:**
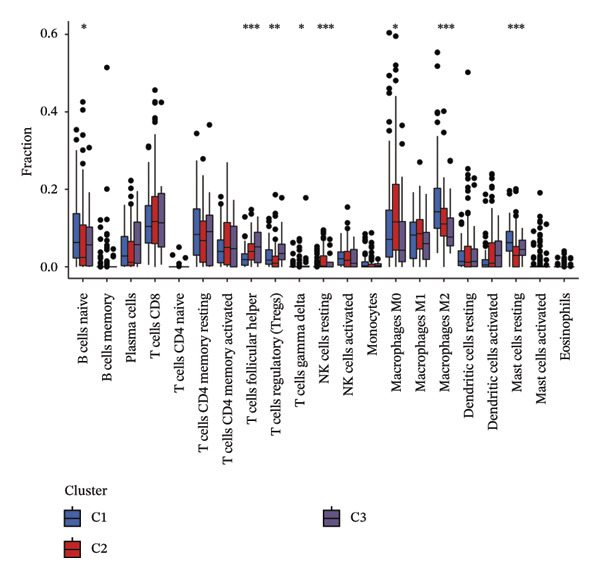
(c)

**Figure:**
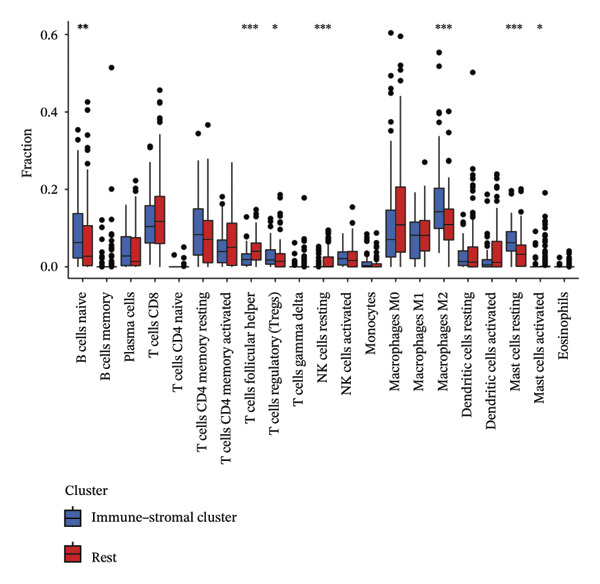
(d)

**Figure:**
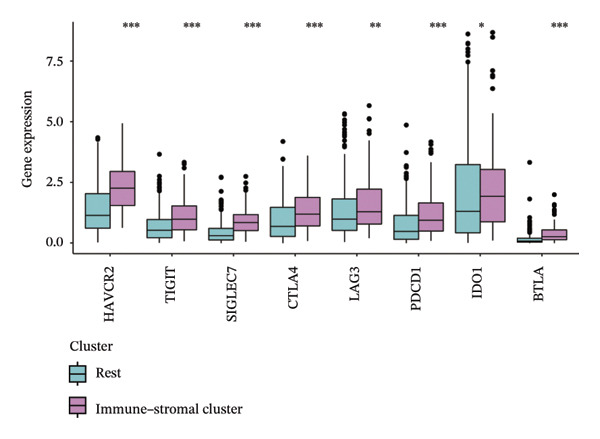
(e)

**Figure:**
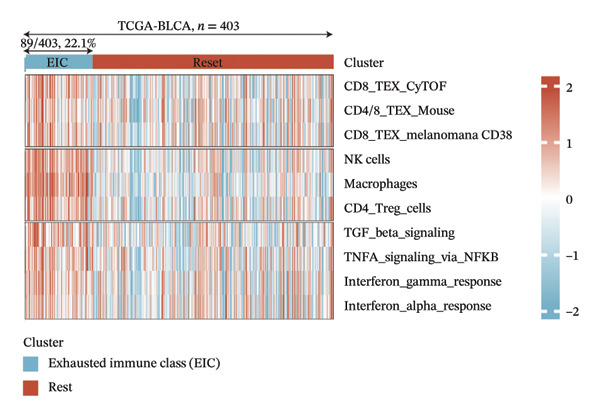
(f)

### 3.6. Molecular Characteristics of EIC

Within the present investigation, Kaplan–Meier survival estimators unveiled that patient cohorts stratified into the EIC subtype exhibited markedly inferior OS outcomes (Figure 6(a)). Additionally, four extra immune‐related signaling pathways—comprising the IMmotion150 effector T cell(Teff) signature, IMmotion150 myeloid cell signature, JAVELIN signature, and tumor inflammation signature (TIS)—were notably suppressed among the EIC subtype (Figure 6(ab)). Mariathasan et al. summarized 21 pathways with predictive value for immunotherapy efficacy, while most of these pathways were inhibited in EIC in our findings (Figure 6(c)). These findings suggest that patients in the EIC cohort may be nonresponsive to ICI therapy. We sought to determine if EIC corresponded to an inflammatory TME phenotype of BLCA and subsequently conducted a comparative analysis of the CIC between these two clusters. As shown in Figure 6(d), most CIC‐mediated functional processes of the EIC subtype were significantly upregulated relative to those of the other cluster, indicating that patients with EIC might enhance tumor‐immune activation and immune cell infiltration of the TME. To further clarify the signaling pathways enriched within EIC, we performed differential gene expression analysis between EIC and Rest clusters, uncovering a total of 2550 differentially expressed genes between the two subgroups (|logFC| ≥ 2, adj.*p* value < 0.05) (Figure 6(e)). GSEA enrichment analysis using HALLMARK gene sets and KEGG gene sets showed that these differentially expressed genes were mainly enriched in cancer‐related pathways, including epithelial–mesenchymal transition pathway, focal adhesion pathway, and E2f target pathway (Figures 6(f), 6(g)).

FIGURE 6Molecular characteristics of EIC. (a) Kaplan–Meier survival estimators revealed that patient cohorts belonging to the EIC subtype exhibited markedly inferior overall survival within the TCGA‐BLCA sample cohort. (b) Via single‐sample gene set enrichment analysis (ssGSEA) and leveraging individual sample transcriptomic expression profiles, the enrichment indices of four supplementary immune‐associated signaling cascades—comprising the IMmotion150 Teff signature, IMmotion150 Myeloid signature, Javelin signature, and TIS (tumor inflammation signature)—were assessed. All four cascades displayed pronounced repression within the EIC subtype. (c) 21 pathways with predictive value for immunotherapy efficacy in the TCGA‐BLCA cohort. (d) We evaluated the activities of these steps using ssGSEA algorithm. Most of the CIC processes of EIC were significantly higher than those of the rest cluster in the TCGA‐BLCA cohort. (e) 2550 differentially expressed genes between EIC and Rest (EIC *vs* Rest) (|logFC| ≥ 2, adj.*p* value < 0.05) in the TCGA‐BLCA cohort. (f–g) GSEA enrichment analysis using HALLMARK gene sets and KEGG gene sets in the TCGA‐BLCA cohort (EIC *vs* Rest; NES: normalized enrichment score) (*p* < 0.05^∗^; *p* < 0.01^∗∗^; *p* < 0.001^∗∗∗^).

**Figure:**
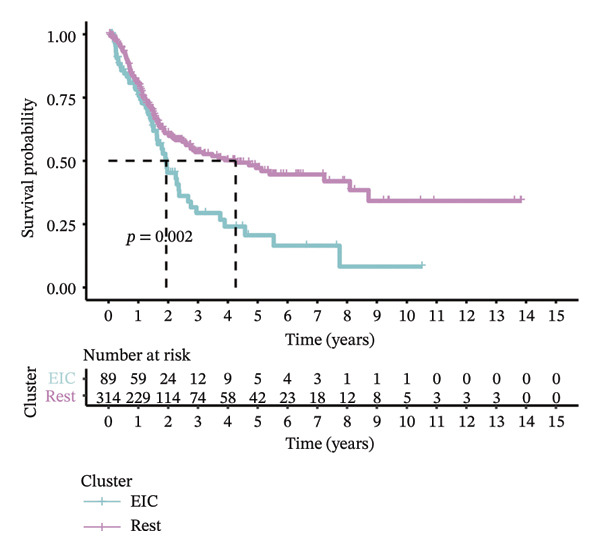
(a)

**Figure:**
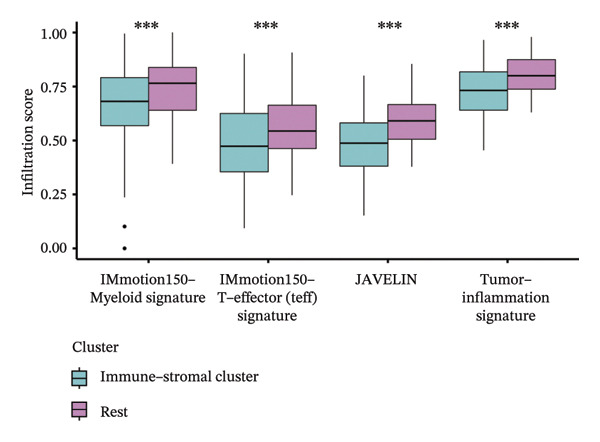
(b)

**Figure:**
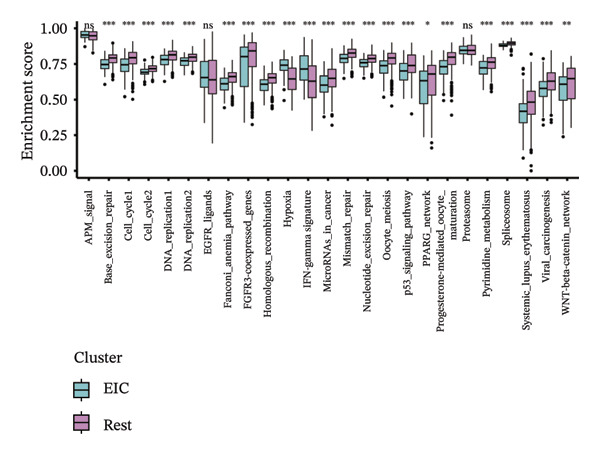
(c)

**Figure:**
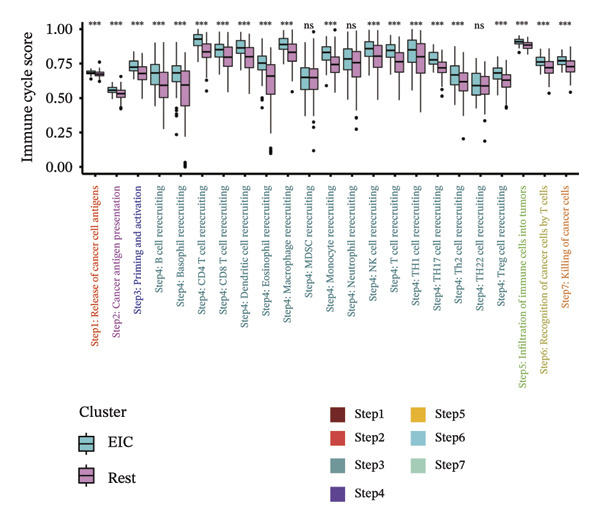
(d)

**Figure:**
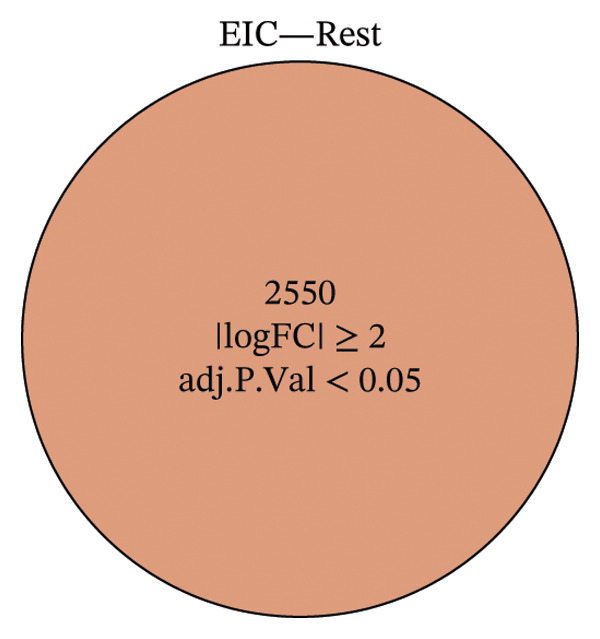
(e)

**Figure:**
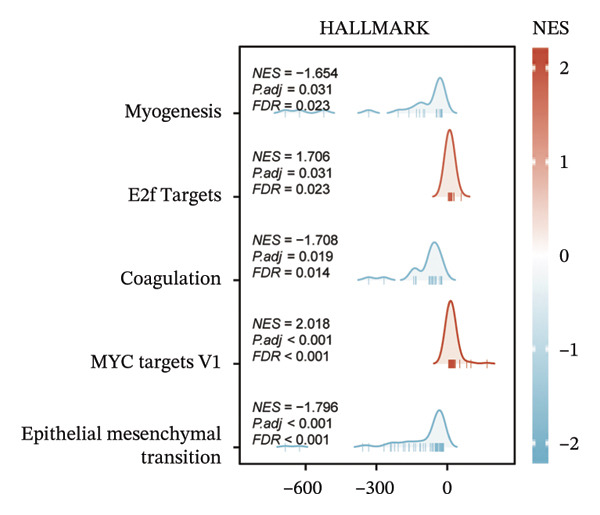
(f)

**Figure:**
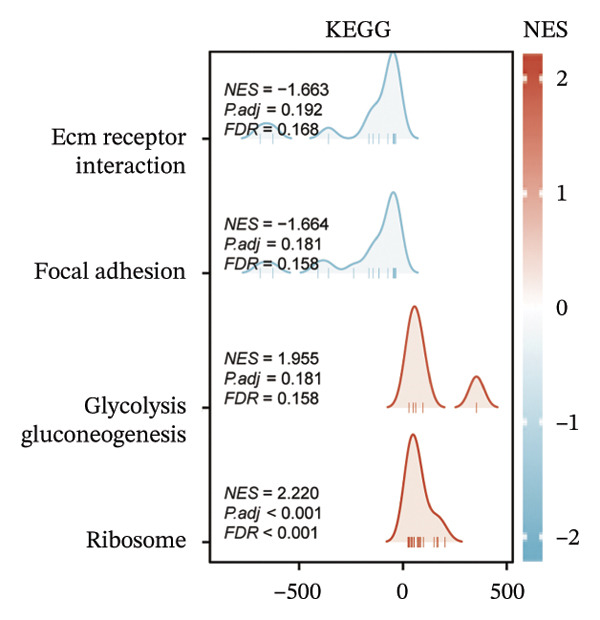
(g)

### 3.7. Immunologic Correlates and Expression Profiles of Hub Genes

We subsequently investigated the linkage between these three hub genes and the tumor‐immune microenvironment. Notably, a marked association was observed between these key genes and immune cell infiltration extent in BLCA, with HSPA12A showing the strongest correlation with central memory T cells (Tcm) and Tem cell infiltration levels (Figure 7(a)). Specifically, BIRC5 exhibited the strongest correlation with Th2 cells and Tgd cells, while CXCL12 showed a pronounced association with macrophages, mast cells, and natural killer (NK) cells (Figures 7(b), 7(c)). Beyond this, a potent positive linear association (*r* > 0.5) was delineated between BIRC5 and CXCL12 transcript abundances and the magnitude of immune cell infiltration. Thereafter, the disparate transcriptional expression profiles of the trio of hub genes were characterized among distinct subgroup classifications. HSPA12A and CXCL12 were found to be highly expressed in the EIC group, whereas BIRC5 displayed elevated expression in the Rest group (Figure 7(d)). We validated these gene expression differences across various cancer types. Notably, BIRC5 was consistently upregulated in most tumors, while HSPA12A and CXCL12 exhibited predominantly low expression levels (Figures 7(e), 7(f), and 7(g)).

FIGURE 7Immunologic correlates and expression profiles of hub genes in the TCGA‐BLCA cohort. (a) HSPA12A was most correlated with the infiltration level of Tcm and Tem cells. (b–c) BIRC5 exhibited the strongest correlation with Th2 cells and Tgd cells, while CXCL12 showed a pronounced association with macrophages, mast cells, and NK cells. (d) HSPA12A and CXCL12 were demonstrated to exhibit markedly elevated expression within the EIC cohort, whereas BIRC5 displayed elevated expression in the Rest group. (e–g) BIRC5 was consistently upregulated in most tumors, while HSPA12A and CXCL12 exhibited predominantly low expression levels (*p* < 0.05^∗^; *p* < 0.01^∗∗^; *p* < 0.001^∗∗∗^).

**Figure:**
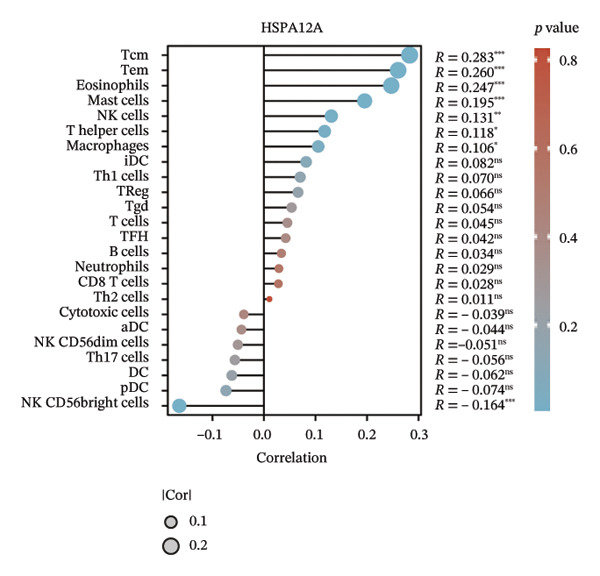
(a)

**Figure:**
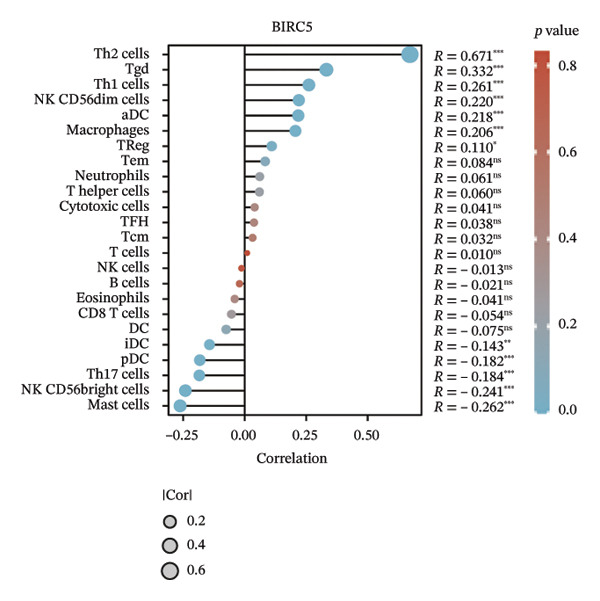
(b)

**Figure:**
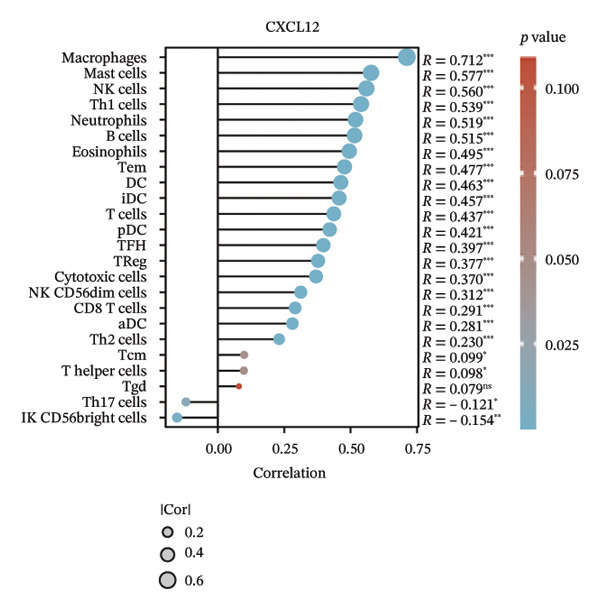
(c)

**Figure:**
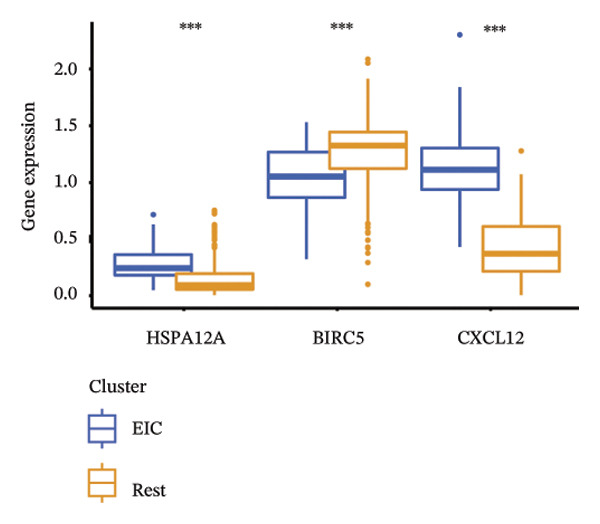
(d)

**Figure:**
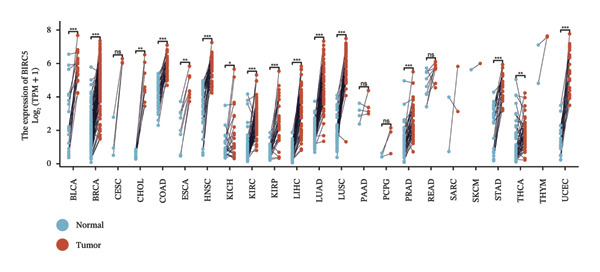
(e)

**Figure:**
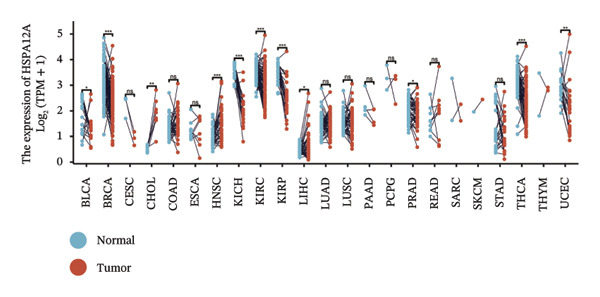
(f)

**Figure:**
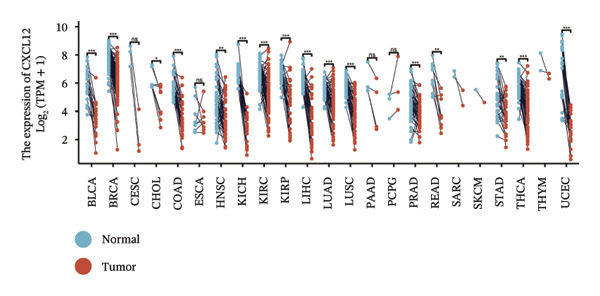
(g)

For BIRC5, we performed a systematic single‐gene analysis. We combine multiple BLCA queue (GSE13507, GSE19423, GSE37815, and TCGA_BLCA) on BIRC and BLCA to explore the relationship between the clinical features. It was found that the high expression of BIRC5 predicted a higher grade and probability of progression in BLCA patients (Figures S3(a) and S3(b)). In addition, high BIRC5 expression in GSE48075, GSE154261, and TCGA_BLCA cohorts predicted poor survival of BLCA patients (Figure S3(c)). BIRC5 was also explored with the use of the IMvigor 210 treatment cohort. Study analysis findings indicated that BIRC5 had a positive linkage with advanced clinical staging among the research population (Figure S4(a)). In addition, subjects exhibiting elevated BIRC5 transcript levels demonstrated superior therapeutic responsiveness to atezolizumab and platinum‐based regimens within the cohort (Figure S4(b)). We next examined the effect of BIRC5 on immunotherapy in the IMvigor 210 (anti‐PD‐L1) treatment cohort and the Wolf cohort 2021 (anti‐PD‐L1) treatment cohort. We reached a consistent conclusion that patients with high BIRC5 expression benefited more from anti‐PD‐L1 treatment (Figure S4(c)). The differential expression of three key genes (BIRC5, HSPA12A, and CXCL12) in BLCA with different molecular subtypes and histological types was also investigated. Among them, BIRC5 showed a completely opposite expression profile to the other two genes (Figures S5(a), S5(b), S5(c), S5(d), S5(e), and S5(f). Finally, we cross‐linked the molecular subtyping of BIRC5, Clusters 1–3, EIC/Rest, and BLCA and explored the distribution of BIRC5 in different subtyping strategies. The results are shown in Figure S5(g).

### 3.8. BIRC5 Is Upregulated in BLCA and Promotes the Proliferation and Invasion of Tumor Cells

Given the unique properties of BIRC5 in bioinformatics analysis, we then explored its possible roles in BLCA initiation and progression by means of a sequence of in vitro experimental trials. We first demonstrated that BIRC5 was upregulated in BLCA tissues, both at the mRNA and protein levels (Figures 8(a) and 8(b)). These findings suggest that elevated BIRC5 expression is closely associated with the pathogenesis of BLCA.

FIGURE 8BIRC5 is up‐regulated in BLCA. We collected tumor tissues and their paired paracarcinoma tissues from six bladder cancer patients and validated the expression of BIRC5. The band density of western blots was calculated by Image J software. The test of each sample was repeated in three independent experiments. BIRC5 was upregulated in BLCA tissues, both at the (a) mRNA and (b) protein levels. (*p* < 0.05^∗^; *p* < 0.01^∗∗^; *p* < 0.001^∗∗∗^, *p* < 0.0001^∗∗∗∗^).

**Figure:**
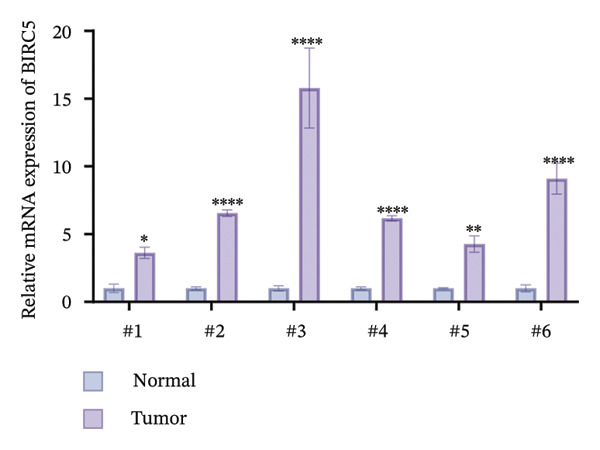
(a)

**Figure:**
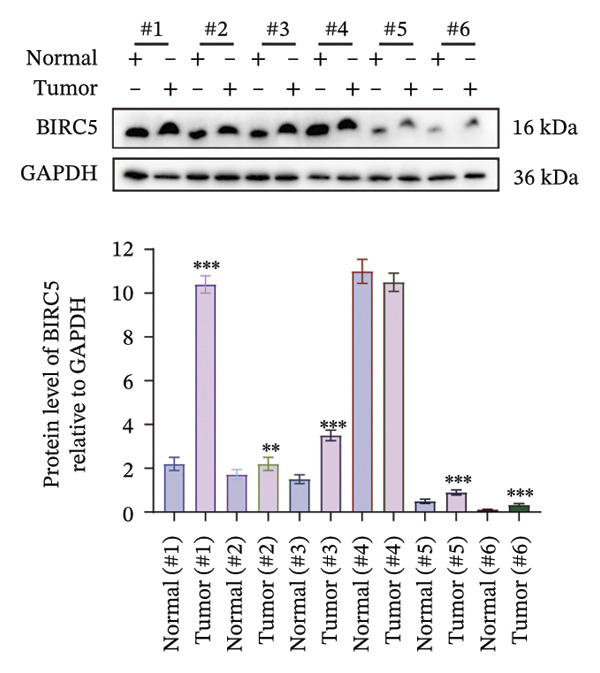
(b)

To decipher the molecular regulatory mechanism of BIRC5 in the oncogenesis and malignant progression of BLCA, targeted knockdown or ectopic overexpression of BIRC5 was implemented in T24 and UMUC3 BLCA cell lines, respectively, and the manipulative efficacy of such genetic interventions was validated (Figures S6(a) and S6(b)). Wound‐healing scratch assay results demonstrated that T24 cell migratory healing capacity declined after BIRC5 knockdown, whereas a reverse effect was seen with BIRC5 overexpression. Similar results were replicated in UMUC3 (Figure 9(a)). Consistently, the results of CCK8 cell viability assay showed that knockdown of BIRC5 inhibited the growth activity of BLCA cells, while the overexpression of BIRC5 increased the cell viability (Figure 9(b)). Transwell‐based detection methods were utilized to assess the migration and invasion capabilities of BLCA cell lines. BIRC5 gene knockdown resulted in a significant decrease in the count of BLCA cells capable of migration and invasion. However, the opposite is true for overexpression (Figure 9(c)). Colony formation assays assessed BLCA cell proliferative capacity, with analogous findings confirming BIRC5’s proproliferative effect on these cells (Figure 9(d)). The above series of in *vitro* experiments proved that BIRC5 could promote the proliferation and migration of BLCA cells.

FIGURE 9BIRC5 promotes the occurrence and development of BLCA. (a) The healing ability of BLCA cells was weakened after the knockdown of BIRC5, while the opposite was observed after the overexpression of BIRC5. (b) CCK8 viability assays revealed that BIRC5 knockdown impaired BLCA cell growth activity, whereas BIRC5 overexpression boosted cell viability. (c) Knockdown of BIRC5 suppressed the number of migrating and invasive BLCA cells. However, the opposite is true for overexpression. (d) Colony formation assays confirmed that BIRC5 could boost BLCA cell proliferation. The original images of the transwell assays are presented in Supporting Figure S8. Each value was measured in triplicate (nc: negative control; si: siRNA; no: negative overexpression; ov: overexpression; *p* < 0.05^∗^; *p* < 0.01^∗∗^; *p* < 0.001^∗∗∗^; *p* < 0.0001^∗∗∗∗^).

**Figure:**
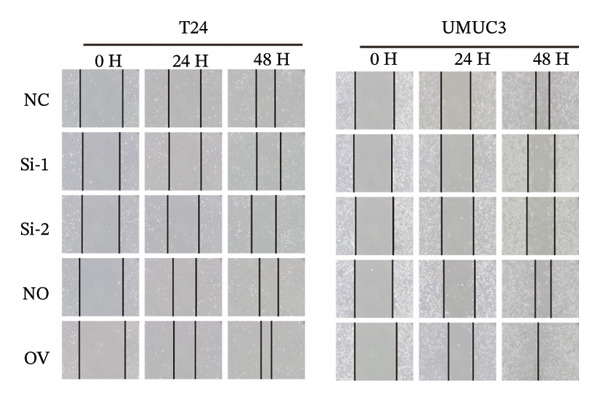
(a)

**Figure:**
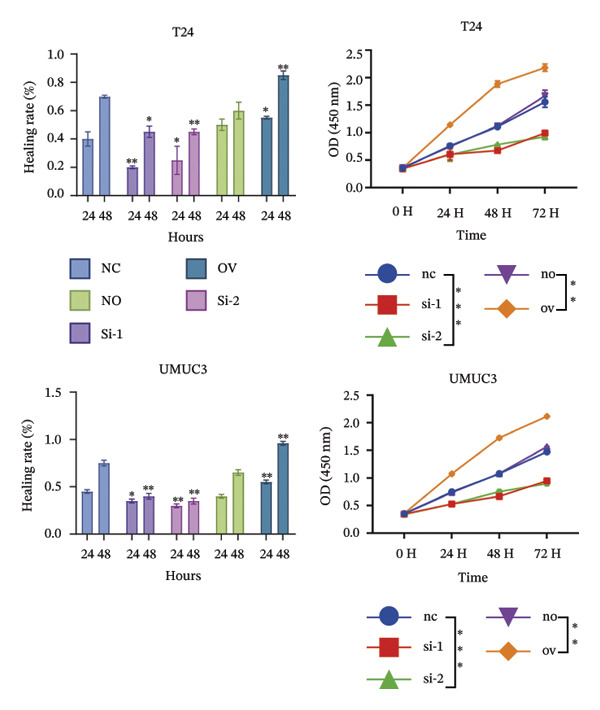
(b)

**Figure:**
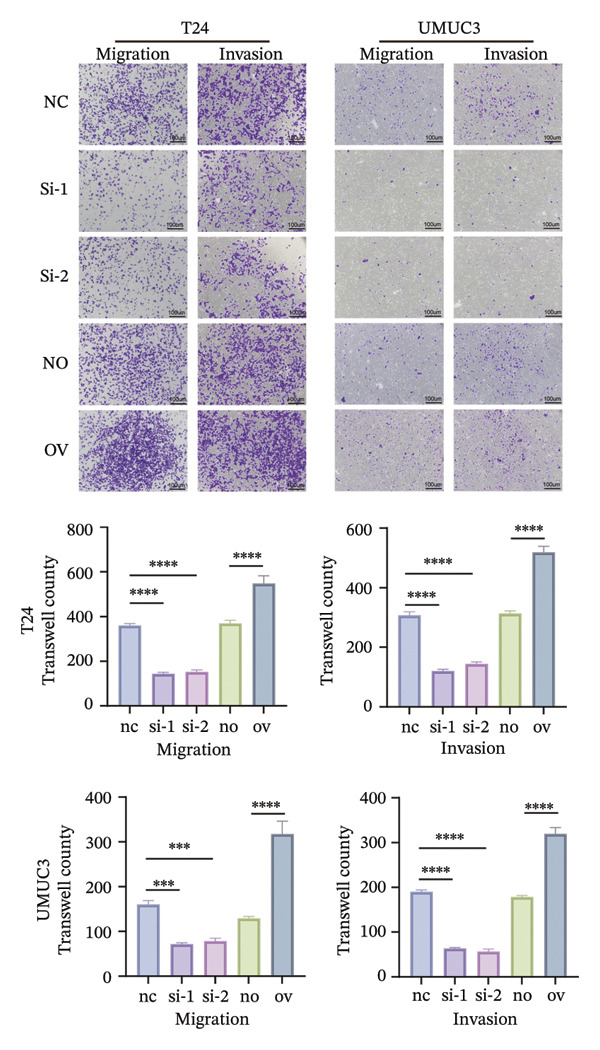
(c)

**Figure:**
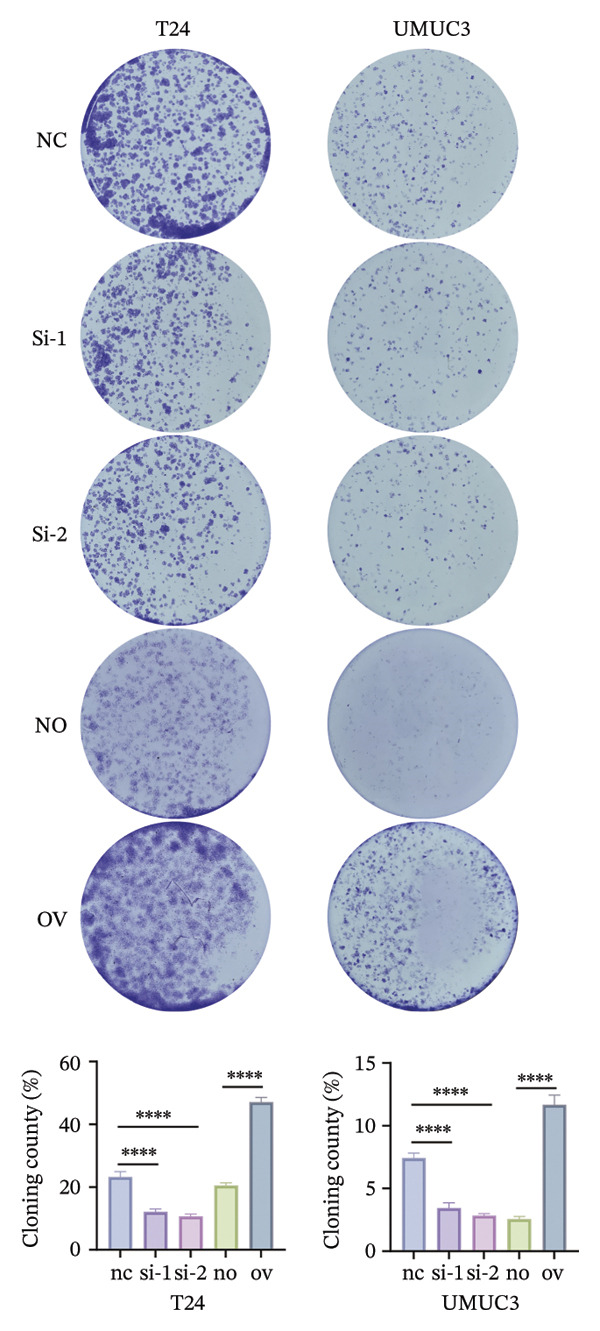
(d)

## 4. Discussion

Currently, therapeutic modalities for BLCA primarily comprise surgery, radiotherapy, chemotherapy, and targeted therapy [11]. Immunotherapy based on immune checkpoint inhibitors, as an emerging treatment method in recent years, has brought new hope to the comprehensive management of BLCA. However, according to clinical data, the immunotherapy response rate of BLCA patients is only about 20% [12]. This highlights the urgency of identifying novel biomarkers and molecular subtypes to optimize immunotherapeutic stratification.

Comprehending the intricate process of tumor immunity is imperative for identifying immunosuppressive targets in cancer screening [13]. The clarification of the CIC has deepened the understanding of antitumor‐immune responses, furnishing a vital opportunity to grasp the dynamic interaction between neoplastic cells and the immune system. Tang’s research team systematically clarified the immune escape pathways within the CIC paradigm, revealing how tumors impede key processes of the cycle to evade immune oversight. [14]. Zhou et al. further validated the practical significance of the CIC by developing a sonoimmune nanotherapy that enhances antitumor efficacy through iterative promotion of key steps in the cycle [15]. Building upon this framework, our study systematically analyzed CICGs in BLCA, identifying BIRC5, CXCL12, and HSPA12A as hub genes and constructing a reliable prognostic model.

Utilizing these three key genes, we conducted molecular subtyping analysis on the TCGA‐BLCA cohort. By examining disparities in the immune microenvironment landscape among distinct subgroups, we successfully discovered a novel immunosuppressive subtype of BLCA (EIC), which accounted for 22.1% of the 403 samples. Bioinformatic analyses confirmed that EIC patients exhibited shortened OS and reduced immunotherapeutic responsiveness [16]. Consistent with findings in hepatocellular carcinoma [17] and lung squamous cell carcinoma [18], our EIC samples showed high enrichment scores for immune cells and stromal components, reflecting their substantial infiltration. EIC specimens were characterized by simultaneous upregulation of immune inhibitory receptors (PD‐1, CTLA‐4, LAG3, etc.), enrichment of immunosuppressive cytokines (IL‐10, TGF‐β, IL‐4), and elevated PD‐L1 expression—features that collectively drive ICB resistance by orchestrating a multilayered immunosuppressive network: metabolic regulation‐induced M2 macrophage polarization amplifies immune evasion [19], CD4+ Treg cells mediate immune escape [20], CD4 T cell dynamics impair combination immunotherapy responses [21], accumulation of immunosuppressive cells reduces PD‐1 blockade efficacy [22], and dysregulated TGF‐β signaling exacerbates ICB resistance [23].

A comprehensive understanding of the molecular characteristics underlying immunosuppressed TME is imperative for reversing tumor‐induced immune suppression and optimizing immunotherapeutic strategies. In our study, we observed elevated expression levels of PD‐1 and CTLA‐4 in tumors from patients with EIC. However, these patients exhibited a limited response to ICB therapy targeting both antibodies. Recent clinical trials have demonstrated that patients harboring multiple factors such as high tumor mutation burden (TMB) and dysregulated TGF‐β‐related signaling pathways may derive substantial benefits from anti‐PD‐1 and anti‐CTLA‐4 antibody treatments. Specifically, Cristescu et al. confirmed via pantumor analyses that high TMB correlates with better responses to anti‐PD‐1 therapy [22]; Derynck et al. further noted that targeting dysregulated TGF‐β signaling can enhance the therapeutic benefit of anti‐CTLA‐4 antibodies [23]. For clinical practice, we propose a stratified strategy: [1] High PD‐L1^+^/EIC^+^ patients—ICI monotherapy is unlikely to be effective, and combination regimens (e.g., ICB + BIRC5 inhibitor + TGF‐β blocker) should be prioritized; [2] high PD‐L1^+^/EIC^-^ patients—ICI monotherapy can be recommended as first‐line treatment; and [3] low PD‐L1^+^/EIC^+^ patients—comprehensive therapy combining chemotherapy, targeted therapy, and immunomodulators may be more appropriate. EIC status can be detected via RNA sequencing or immunohistochemistry targeting core markers (HSPA12A, CXCL12, PD‐1, and CTLA‐4), facilitating its translation into clinical practice. Additional mechanistic inquiries are merited to unravel the associated intrinsic regulatory mechanism.

Subsequently, a comprehensive investigation of the three hub genes revealed that BIRC5 exhibited close associations with immune cells such as Th2 cells and macrophages within the TME, displaying high expression levels in various cancers, including BLCA. According to the existing literature, BIRC5 is widely acknowledged as an antiapoptotic molecule that facilitates cell division and tumor progression, thus being recognized as a potential therapeutic target. Specifically, Frazzi identified BIRC5’s dual role in inhibiting apoptosis and promoting cell division in cancer [24]; consistent with Altieri’s research [25], BIRC5 activates the PI3K/Akt signaling pathway to inhibit M2 macrophage apoptosis, thereby promoting the accumulation of immunosuppressive macrophages in the TME—this amplifies the immunosuppressive landscape that favors tumor escape. Furthermore, it has been reported that BIRC5 orchestrates the aggravation of multiple cancers both in vitro and in vivo by modulating the tumor‐immune microenvironment. Notably, Altieri highlighted BIRC5’s involvement in reshaping immune microenvironment dynamics [25]; Xu et al. validated this effect by showing that BIRC5 modulates immune infiltration to exacerbate cancer progression in preclinical models [26].

Despite some studies suggesting that BIRC5 promotes tumor progression in BLCA, its precise role in BLCA pathogenesis and regulation of the tumor‐immune microenvironment remains elusive. Take for example, Liu et al. documented that ambient fine particulate matter triggers BIRC5‐associated m6A epigenetic modification to facilitate BLCA malignant progression [27]; Yang et al. found that BIRC5 promotes BLCA cell proliferation by targeting miR‐138‐5p [28]—yet neither clarified its specific role in BLCA immune microenvironment regulation. The results of our research confirm excessive expression of BIRC5 in bladder urothelial carcinoma, along with its ability to boost the proliferation, migration, and invasion potential of BLCA cell lines in laboratory in vitro environments. Such data offer valuable insights for deciphering the functional mechanism through which BIRC5 acts in BLCA.

While the TCGA‐BLCA cohort (403 samples covering diverse stages, grades, and treatment backgrounds) ensures basic representativeness, it lacks data on prior immune therapy exposure, restricting the generalization of our results to ICI‐treated patients. Future work will validate EIC’s value in prospective cohorts with detailed immunotherapy records, alongside three targeted trials: Phase II (ICB + BIRC5 inhibitor + TGF‐β blocker in EIC^+^ patients), Phase III (stratified by EIC status in high PD‐L1^+^ patients), and neoadjuvant trial (BIRC5 inhibitor in resectable BLCA) of EIC/BIRC5‐targeted regimens, to confirm utility for immunotherapy‐resistant BLCA.

## 5. Conclusion

After conducting a comprehensive analysis, we have successfully characterized the molecular attributes of CICGs in BLCA for the first time. Furthermore, we have identified and screened out the core genes that are closely associated with BLCA. Moreover, by effectively classifying BLCA patients based on key CICGs, we have discovered a distinct population exhibiting unique EIC subtypes. These findings provide novel insights into personalized immunotherapy.

### 5.1. Limitation

Although we identified BIRC5 as a prognostic CIC gene in BLCA, the immunophenotypic association of BIRC5 with BLCA was not demonstrated in this study. The “exhausted immune” phenotype of the identified “exhausted immune class” of BLCA remains to be validated. We are currently conducting a systematic study on the immune correlation between BIRC5 and BLCA, looking forward to providing new insights into the immunotherapy of BLCA [29].

## Author Contributions

Zexun Deng, Yifan Li, Yuyong Shen, and Ming Zhou undertook data analyses and drafted the initial manuscript. Ming Zhou and Zexun Deng retrieved and acquired raw datasets from the TCGA and GEO public databases. Ming Zhou, Yifan Li, and Yuyong Shen participated in specialized statistical analyses. Zexun Deng and Yifan Li completed the visualization and editing of experimental figures. Yifan Li and Ming Zhou formulated the overall research framework and supervised the entire research workflow. All contributors participated in the reviewing and polishing of the manuscript. Each author made significant contributions to the final version of the article.

## Funding

The present research undertaking obtained funding support from the Social Development Program of the Yangzhou Municipal Bureau of Science and Technology (Grant No. YZ2023080).

## Disclosure

All authors approved the officially submitted manuscript.

## Ethics Statement

This research obtained ethical clearance from the Institutional Ethics Committee of the Affiliated Hospital of Yangzhou University and was carried out in adherence to the ethical protocols outlined in the 1964 Declaration of Helsinki, its updated iterations, and corresponding ethical criteria. The designated ethics review authorization code was 2025‐YKL10‐K10.

## Conflicts of Interest

The authors declare no conflicts of interest.

## Supporting Information

Additional supporting information can be found online in the Supporting Information section.

## Supporting information


**Supporting Information 1** Figure S1: Prognostic risk of the 11 DECICGs in the TCGA‐BLCA cohort. (a) Correlations and prognosis of the 11 DECICGs in patients with BLCA. (b) Five of the 11 DECICGs showed significant correlation with poor overall survival of patients with BLCA.

**Supporting Information 2** Figure S2: Screening of hub DECICGs via WGCNA in GSE166716. (a–c) The same analysis method as TCGA‐BLCA was applied again. (d) Twelve color modules were identified in the GSE166716 cohort. (e) The green module (*r* = 0.79, *p* < 0.001) was positively correlated with BLCA, whereas the turquoise module (*r* = −0.95, *p* < 0.001) was negatively correlated with BLCA. (f) MM was significantly correlated with the GS of the green module. (g) BIRC5 and EZH2 were present in the green module, while KLF2, HSPA12A, ARG2, EDNRB, HSPA2, CXCL12, and CCL19 were present in the turquoise module.

**Supporting Information 3** Figure S3: Clinical relevance of BIRC5 in multiple BLCA datasets. (a) Interrelation between BIRC5 expression, Subtyping 1–3, EIC subtyping, and BLCA molecular subtyping. (b) BIRC5 expression is positively correlated with the progression of BLCA. (c) BIRC5 is a risk factor for BLCA survival.

**Supporting Information 4** Figure S4: Correlation between BIRC5 and response to targeted therapy and immunotherapy in BLCA. (a) In the IMvigor210 cohort, patients with higher BIRC5 expression had higher tumor stage. (b) In the IMvigor210 cohort, patients with higher BIRC5 expression had better treatment response to atezolizumab and platinum. (c) The effect of BIRC5 on immunotherapy in the IMvigor 210 (anti‐PD‐L1) treatment cohort and the Wolf cohort 2021 (anti‐PD‐L1) treatment cohort. The ROC curve showed the immunotherapy response prediction.

**Supporting Information 5** Figure S5: Interrelation between BIRC5 expression, Clusters 1–3, EIC/Rest subtyping, and BLCA molecular clusters. (a–f) BIRC5 showed a completely opposite expression profile to the other two genes. (g) Cross‐link of the molecular subtyping of BIRC5, Clusters 1–3, EIC/Rest, and BLCA, and explored the distribution of BIRC5 in different subtyping strategies.

**Supporting Information 6** Figure S6: Verification of the efficiency of BIRC5 knockdown. (a) qRT‐PCR verification of knocked down or overexpressed BIRC5 in T24 cells and UMUC3 cells. (b) Western blot verification of knocked down or overexpressed BIRC5 in T24 cells and UMUC3 cells. The original blots/gels are presented in Supporting Figure S7. The test of each sample was repeated in three independent experiments (nc: negative control; si: siRNA; no: negative overexpression; ov: overexpression; *p* < 0.05^∗^; *p* < 0.01^∗∗^; *p* < 0.001^∗∗∗^).

**Supporting Information 7** Figure S7: The original gels/blots of Figures​ 8(a) and 8(b).

**Supporting Information 8** Figure S8: The original images of Figure 9(c).

**Supporting Information 9** Table S1: Clinical specimen information.

**Supporting Information 10** Table S2: BIRC5 siRNA sequences and primer sequence for BIRC5.

## Data Availability

Original empirical findings produced during the course of this investigative project have been integrated into the primary manuscript and accompanying supporting data repositories. Further research‐associated queries may be directed to the appointed corresponding author.
