# Supplementary figures and images for: Comprehensive Analysis Based on the Cancer‐Immunity Cycle Identifies a Novel Immunosuppressive Subtype of Bladder Cancer
Source: Int J Genomics. 2026 Apr 6;2026:9720283. doi: 10.1155/ijog/9720283 (PMC13051804; doi:10.1155/ijog/9720283)

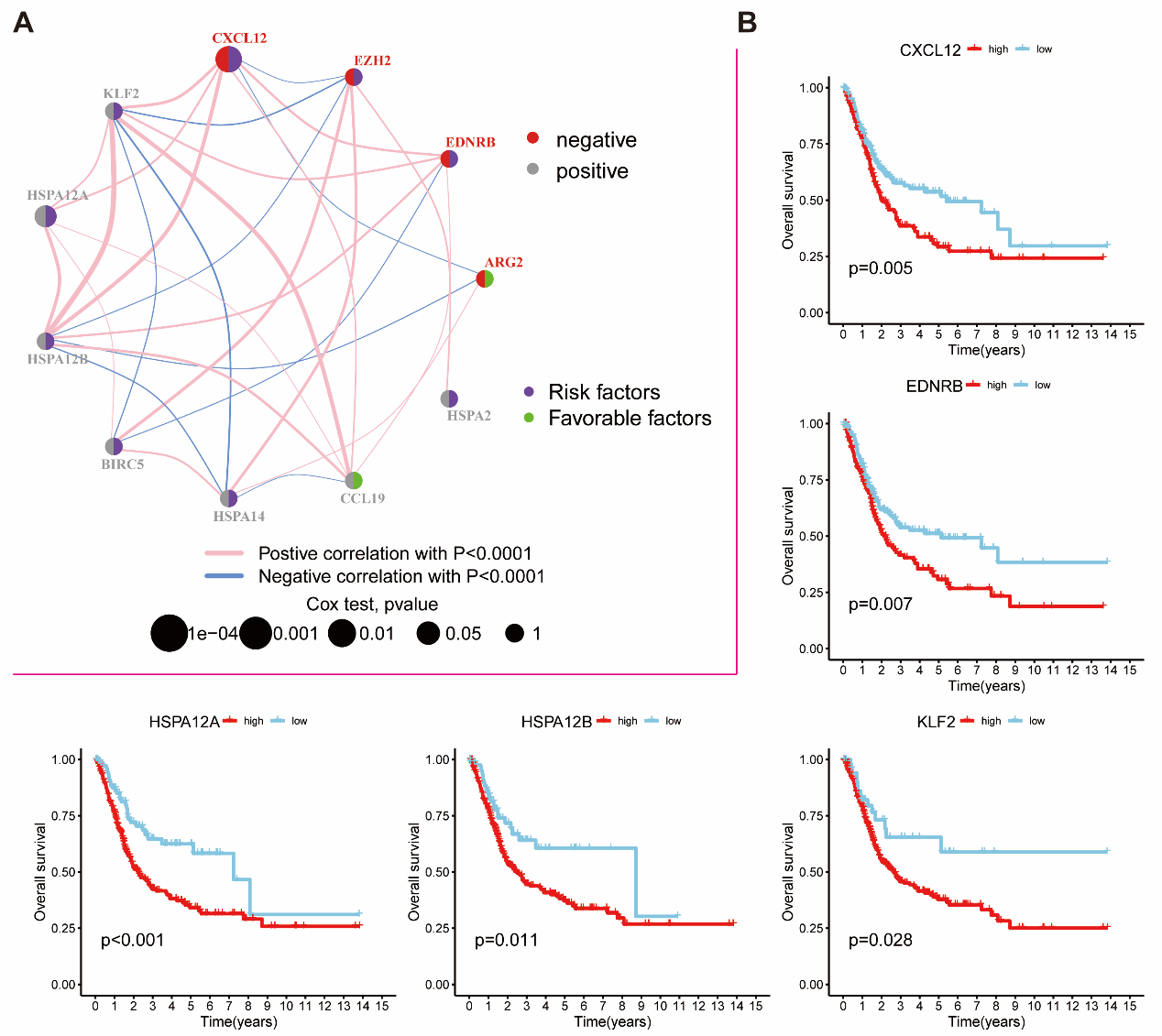

Supplement: Supplementary file 1 — Supporting Information 1 Figure S1: Prognostic risk of the 11 DECICGs in the TCGA‐BLCA cohort. (a) Correlations and prognosis of the 11 DECICGs in patients with BLCA. (b) Five of the 11 DECICGs showed significant correlation with poor overall survival of patients with BLCA. [file IJOG-2026-9720283-s002.png]

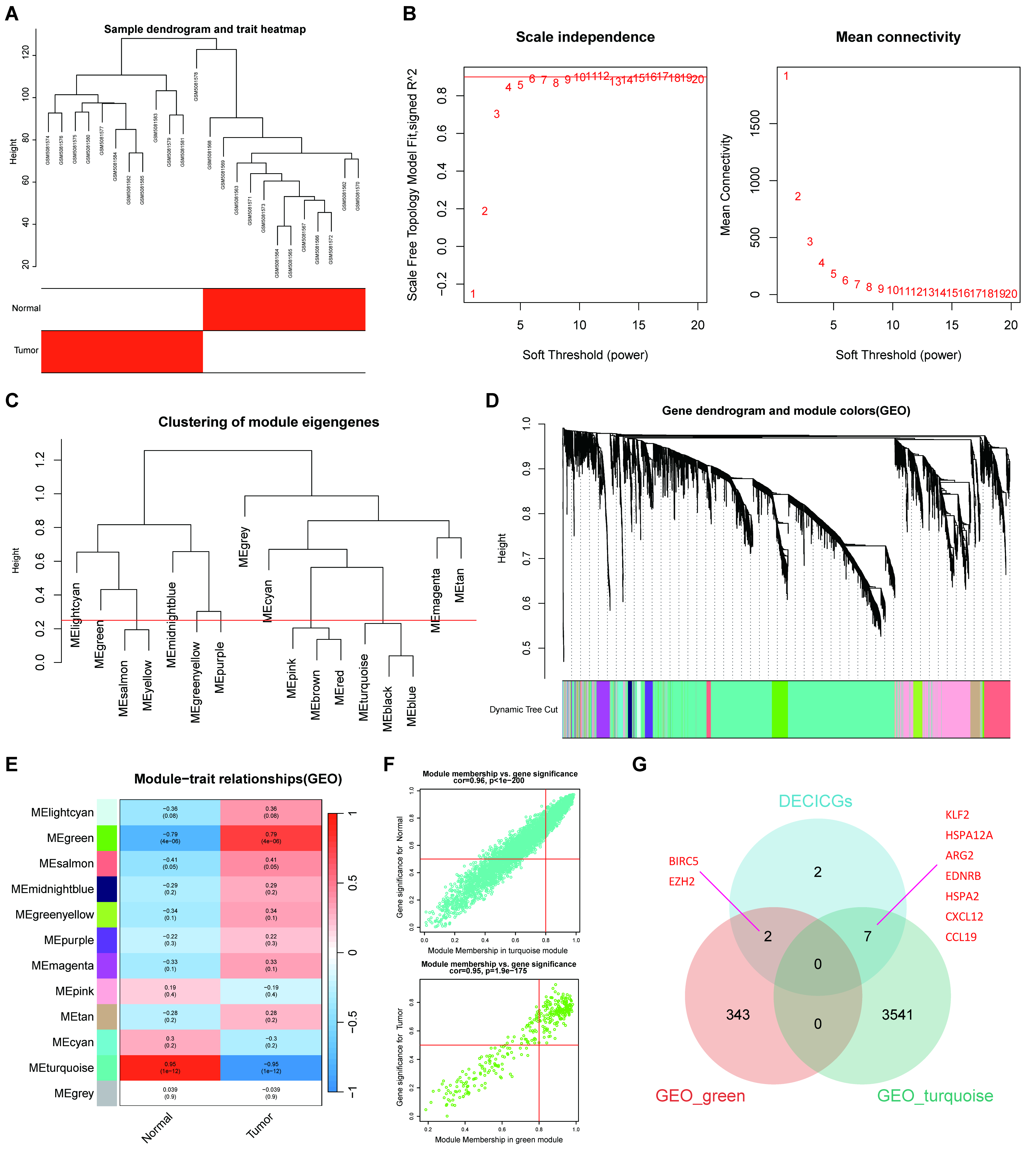

Supplement: Supplementary file 2 — Supporting Information 2 Figure S2: Screening of hub DECICGs via WGCNA in GSE166716. (a–c) The same analysis method as TCGA‐BLCA was applied again. (d) Twelve color modules were identified in the GSE166716 cohort. (e) The green module (r = 0.79, p < 0.001) was positively correlated with BLCA, whereas the turquoise module (r = −0.95, p < 0.001) was negatively correlated with BLCA. (f) MM was significantly correlated with the GS of the green module. (g) BIRC5 and EZH2 were present in the green module, while KLF2, HSPA12A, ARG2, EDNRB, HSPA2, CXCL12, and CCL19 were present in the turquoise module. [file IJOG-2026-9720283-s003.png]

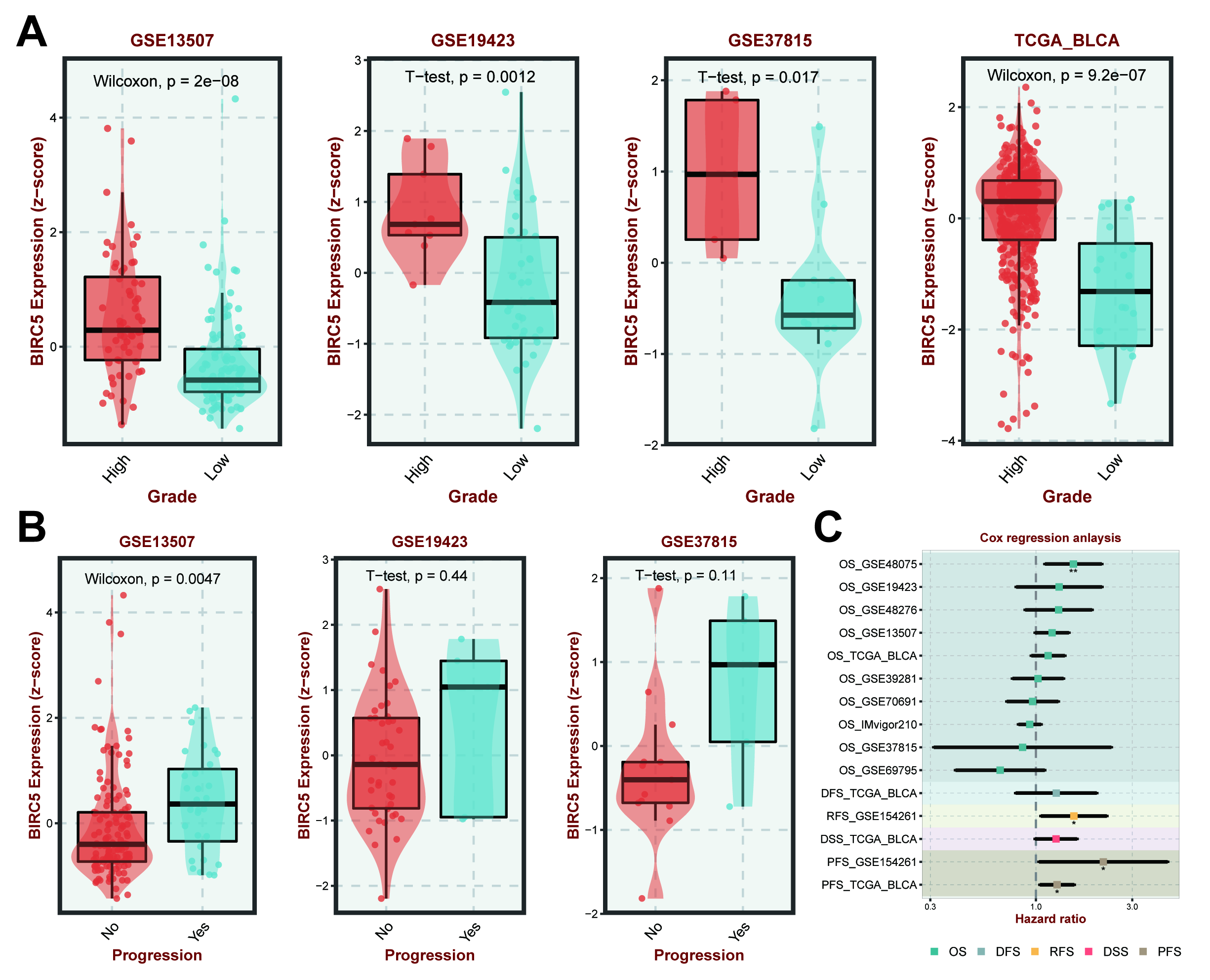

Supplement: Supplementary file 3 — Supporting Information 3 Figure S3: Clinical relevance of BIRC5 in multiple BLCA datasets. (a) Interrelation between BIRC5 expression, Subtyping 1–3, EIC subtyping, and BLCA molecular subtyping. (b) BIRC5 expression is positively correlated with the progression of BLCA. (c) BIRC5 is a risk factor for BLCA survival. [file IJOG-2026-9720283-s004.png]

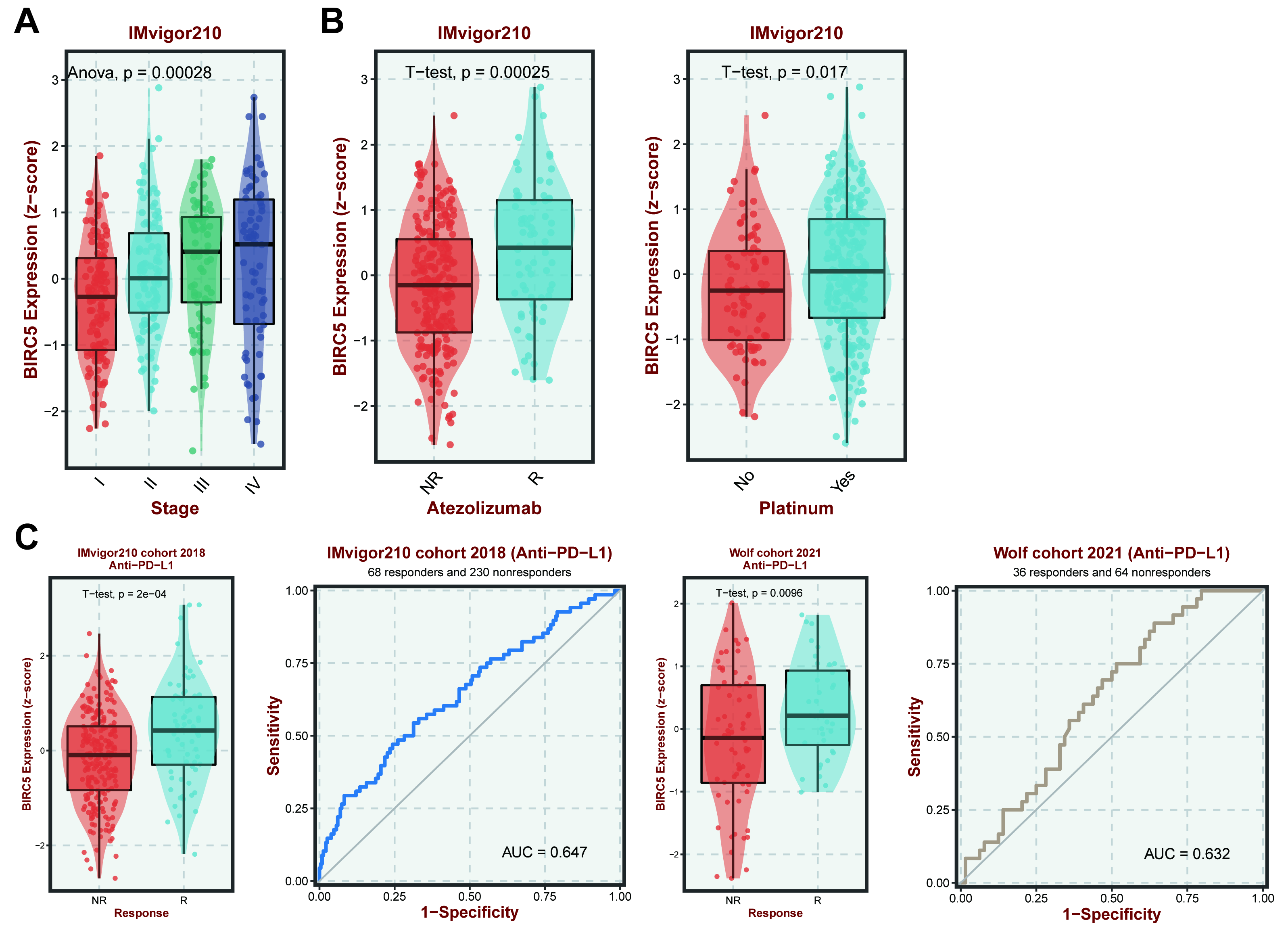

Supplement: Supplementary file 4 — Supporting Information 4 Figure S4: Correlation between BIRC5 and response to targeted therapy and immunotherapy in BLCA. (a) In the IMvigor210 cohort, patients with higher BIRC5 expression had higher tumor stage. (b) In the IMvigor210 cohort, patients with higher BIRC5 expression had better treatment response to atezolizumab and platinum. (c) The effect of BIRC5 on immunotherapy in the IMvigor 210 (anti‐PD‐L1) treatment cohort and the Wolf cohort 2021 (anti‐PD‐L1) treatment cohort. The ROC curve showed the immunotherapy response prediction. [file IJOG-2026-9720283-s010.png]

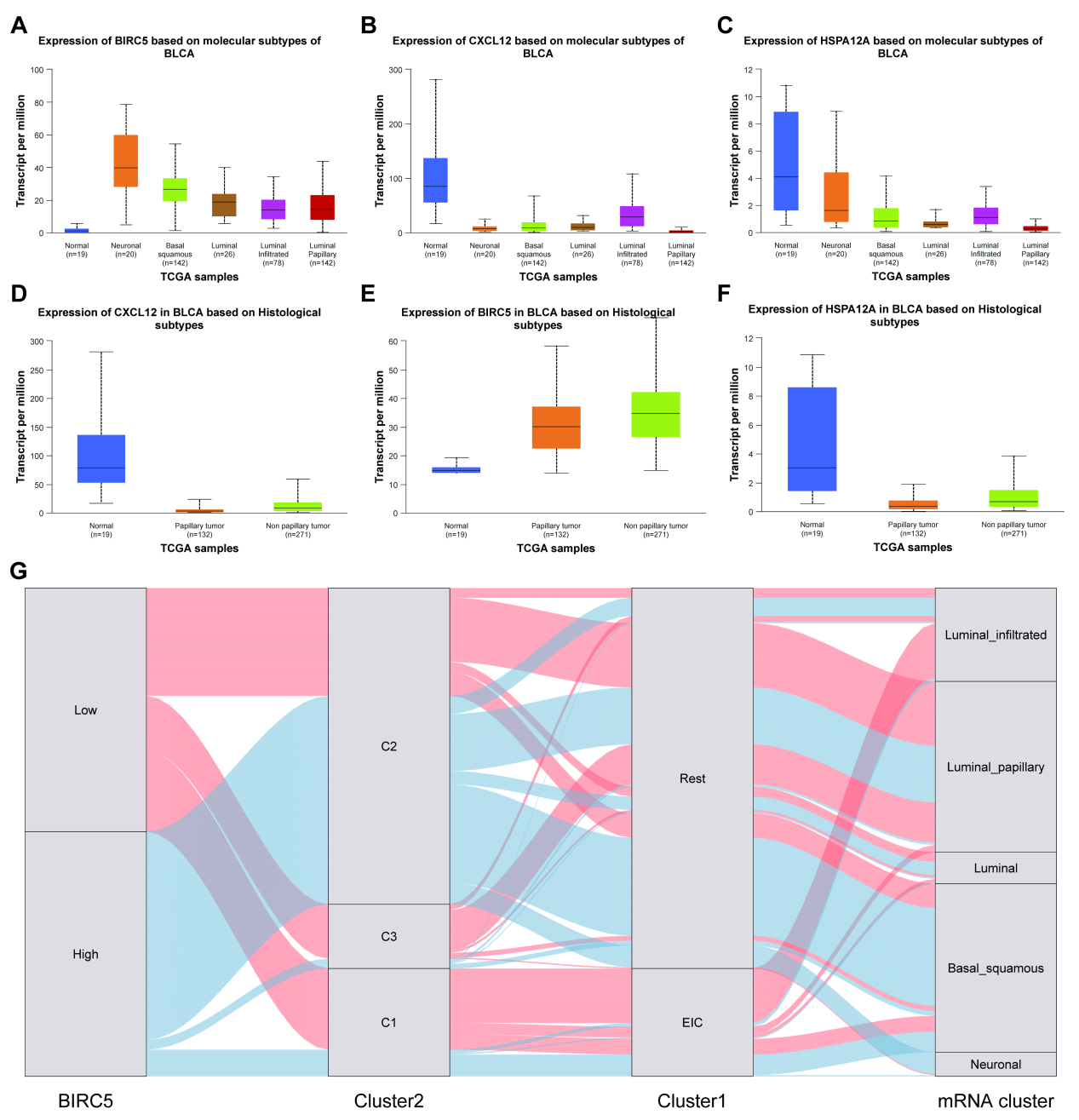

Supplement: Supplementary file 5 — Supporting Information 5 Figure S5: Interrelation between BIRC5 expression, Clusters 1–3, EIC/Rest subtyping, and BLCA molecular clusters. (a–f) BIRC5 showed a completely opposite expression profile to the other two genes. (g) Cross‐link of the molecular subtyping of BIRC5, Clusters 1–3, EIC/Rest, and BLCA, and explored the distribution of BIRC5 in different subtyping strategies. [file IJOG-2026-9720283-s006.png]

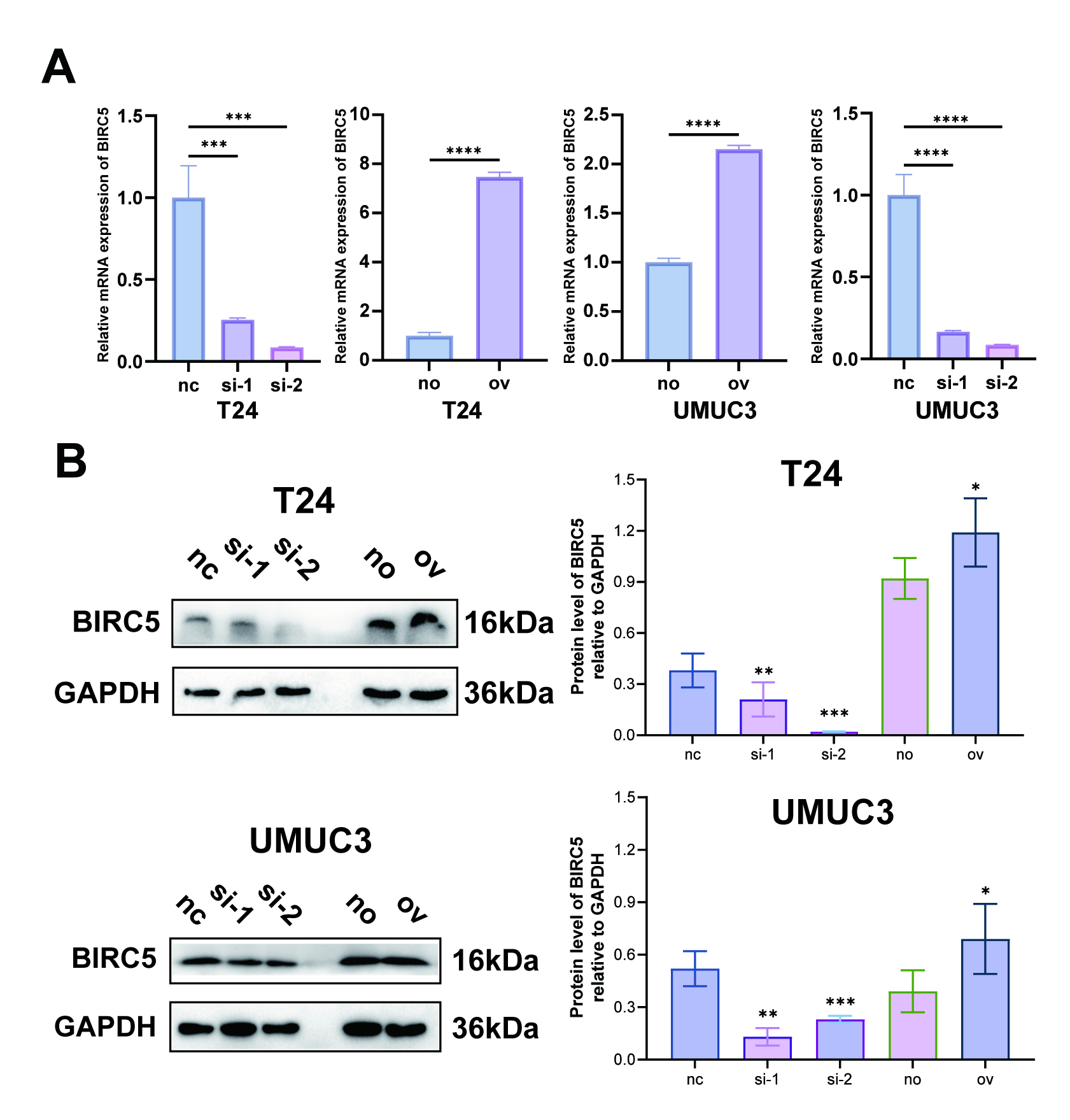

Supplement: Supplementary file 6 — Supporting Information 6 Figure S6: Verification of the efficiency of BIRC5 knockdown. (a) qRT‐PCR verification of knocked down or overexpressed BIRC5 in T24 cells and UMUC3 cells. (b) Western blot verification of knocked down or overexpressed BIRC5 in T24 cells and UMUC3 cells. The original blots/gels are presented in Supporting Figure S7. The test of each sample was repeated in three independent experiments (nc: negative control; si: siRNA; no: negative overexpression; ov: overexpression; p < 0.05∗; p < 0.01∗∗; p < 0.001∗∗∗). [file IJOG-2026-9720283-s007.png]

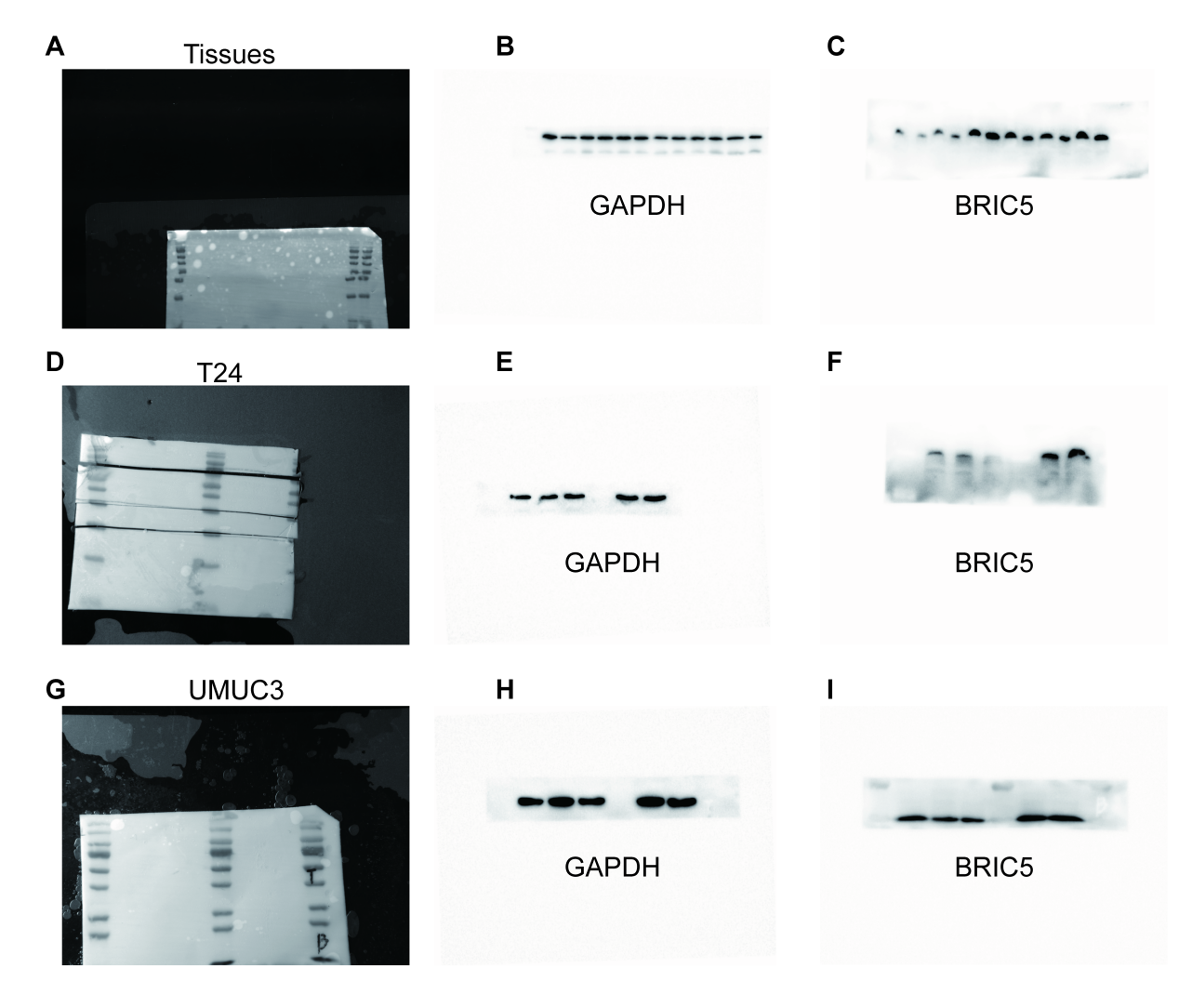

Supplement: Supplementary file 7 — Supporting Information 7 Figure S7: The original gels/blots of Figures​ 8(a) and 8(b). [file IJOG-2026-9720283-s008.png]

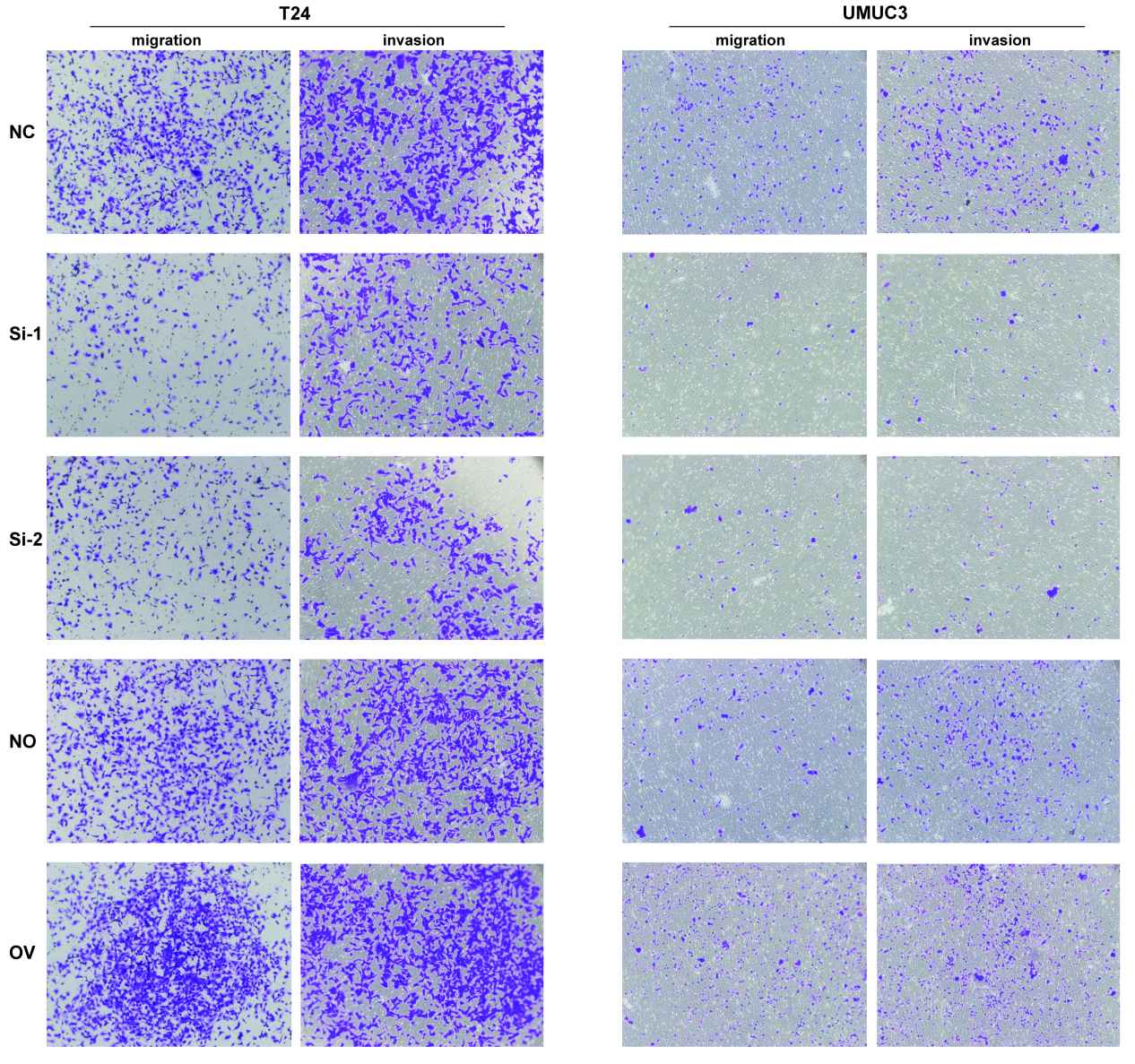

Supplement: Supplementary file 8 — Supporting Information 8 Figure S8: The original images of Figure 9(c). [file IJOG-2026-9720283-s009.png]
